# Microengineering the Liver: Strategies for Constructing Functional Liver‐on‐a‐Chip Devices

**DOI:** 10.1002/exp2.70137

**Published:** 2026-03-03

**Authors:** Jie Wang, Ziwei Liang, Jiapu Wang, Zongyi Li, Shaojie Wang, Yan Wei, Xin Xie, Di Huang

**Affiliations:** ^1^ Department of Biomedical Engineering Research Center for Nano‐Biomaterials & Regenerative Medicine College of Artificial Intelligence Shanxi Key Laboratory of Materials Strength & Structural Impact Taiyuan University of Technology Taiyuan China; ^2^ NHC Key Laboratory of Glycoconjuates Research, Department of Biochemistry and Molecular Biology School of Basic Medical Sciences Fudan University Shanghai China; ^3^ Shanxi‐Zheda Institute of Advanced Materials and Chemical Engineering Taiyuan China; ^4^ Shanxi Provincial Key Laboratory for Functional Proteins Shanxi Jinbo Bio‐Pharmaceutical Co, Ltd Taiyuan China; ^5^ Xellar‐Biosystems Cambridge Massachusetts USA

**Keywords:** deep learning, disease modeling, drug screening, liver‐on‐a‐chip, microphysiological systems

## Abstract

Reliable in vitro liver models are indispensable for researching liver diseases and developing medications. Present 2D/3D cell cultures and animal models inadequately replicate the intricacy of living systems and in vivo conditions, resulting in impaired cellular functions. They also fail to emulate tissue‐like architectures, which undermines their precision. Meanwhile, animal models present species differences, making real‐time observation of dynamic results inconvenient and raising serious ethical concerns. Therefore, there is an urgent need to develop alternative tissue models with biomimetic human pathophysiology to bridge the gap between clinical trials and traditional human and animal models. Liver‐on‐a‐chip (LOC) technology, based on microfluidics, is an innovative in vitro modeling device that can replicate the microstructures and tissue‐tissue interfaces of specific liver functional units, simulating organ and tissue‐level physiological activities. This review summarizes recent strategies and breakthroughs in LOC technologies, from biomimetic tissues and extracellular matrix construction in liver microphysiological systems to diverse LOC development approaches. Furthermore, we highlight key advances in functional LOC platforms, including 3D bioprinting, vascularization strategies, and the incorporation of liver buds and organoids to enhance physiological relevance. The integration of deep learning and sensor technologies for intelligent, real‐time monitoring is also discussed. Finally, we examine LOC applications in drug screening and disease modeling, assess challenges in clinical translation, and offer perspectives on future directions in biomedical research and personalized medicine.

## Graphical abstract

This review summarizes recent advances in liver‐on‐a‐chip (LOC) technologies, including fabrication strategies and functional integration approaches, and discusses their promising applications in drug screening and related biomedical fields.

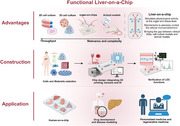

## 1 Introduction

The liver, the most voluminous and functionally diverse organ in the human body, plays a pivotal role in maintaining homeostasis and executing various vital physiological processes [1]. Current liver research and drug toxicity testing predominantly rely on 2D or simple 3D cell culture models and animal models [2]. However, 2D or simple 3D cell culture models lack the complexity of living systems, fail to replicate the in vivo physiological environment, and may result in cellular function loss due to the absence of cell‐cell and cell‐matrix interactions. Moreover, these models cannot control cell spatial arrangements and typically lack three‐dimensional tissue‐like microstructures, severely compromising their physiological accuracy and reliability in reconstructing tissue‐specific functions and microenvironmental characteristics [3]. At the same time, animal models suffer from species differences, are inconvenient for real‐time observation of dynamic results, and raise serious ethical concerns. Consequently, there is an urgent need for alternative tissue models with bionic human pathophysiology to bridge the gap between clinical trials and conventional models.

**FIGURE 1:**
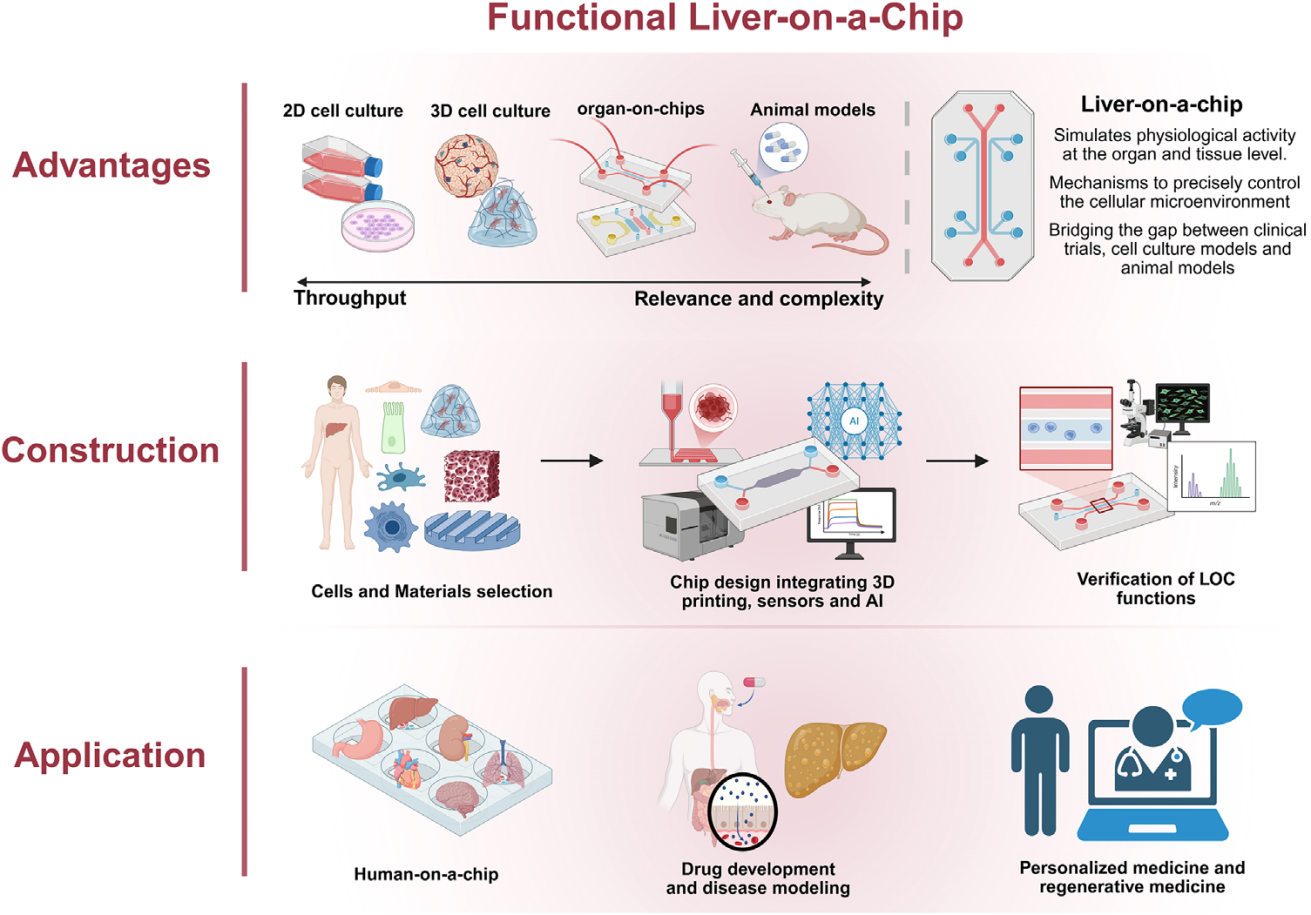
Advantages, efficient construction, and applications of LOC (Created in BioRender).

Microfluidic‐based organ chips are innovative in vitro modeling devices that replicate the microstructure and tissue‐tissue interfaces of specific organ functional units, simulating physiological activities at the organ and tissue levels [4, 5]. The first in vitro biomimetic system to emulate human organ and tissue physiology by incorporating cells within a microfluidic chip was published by Michael Shuler et al. in 2004 [6, 7]. Since then, researchers have developed kidney‐on‐a‐chip [8], vascular‐on‐a‐chip [9], lung‐on‐a‐chip [10, 11, 12], and bone‐on‐a‐chip [13] using tissue engineering, microfluidics, and microengineering techniques. Donald Ingber et al. introduced the term “organ‐on‐a‐chip (OoC)” in 2010 [11]. The emergence of liver‐on‐a‐chip (LOC) has broadened the prospects for liver physiology and pathology studies as well as drug toxicity testing. This study systematically reviews advancements ranging from tissue and extracellular matrix biomimetics in liver microphysiological systems to LOC platforms enhanced by deep learning and 3D bioprinting technologies, with a particular focus on the latest strategies and technological breakthroughs in LOC construction. It investigates approaches for functional enhancement and explores their application value in drug screening, disease modeling, and the development of human‐on‐a‐chip systems. Furthermore, this review provides an in‐depth analysis of the advantages and challenges associated with the practical implementation of LOC technologies and offers a forward‐looking perspective on their future development (Figure 1).

## 2 Liver‐on‐a‐Chip

### 2.1 Components of LOC

#### 2.1.1 LOC Manufacturing‐Cell Source

The liver comprises a diverse array of cell types, broadly categorized into parenchymal and non‐parenchymal cells. Hepatocytes (HCs), the primary parenchymal cells, are the most abundant cell type in the liver and are mainly responsible for metabolism, detoxification, protein synthesis, and bile production. In contrast, non‐parenchymal cells include cholangiocytes, hepatic stellate cells (HSCs), Kupffer cells (KCs), and liver sinusoidal endothelial cells (LSECs). Although fewer in number, non‐parenchymal cells are essential for maintaining liver homeostasis. HSCs are involved in liver repair and fibrosis, KCs function in immune surveillance and phagocytosis, LSECs regulate blood filtration and flow, and cholangiocytes contribute to bile modification and transport. Ensuring a physiologically relevant combination of both parenchymal and non‐parenchymal cell types, along with optimal culture conditions, is crucial for sustaining specific liver functions on lab‐on‐a‐chip (LOC) platforms. Currently, the commonly used sources of parenchymal cells in LOC systems are summarized in Table 1, while the sources of non‐parenchymal cells are summarized in Table 2 [14].

**TABLE 1:** Advantages and limitations of the parenchymal cells involved in the LOC.

Cell type	Advantages	Limitations
Primary human hepatocytes	Closest to in vivo liver function; High enzymatic activity; Robust synthetic and metabolic function	Loss of specific functions; Poor long‐term viability; Limited donor availability; Technically demanding isolation
Stem cell induces hepatocytes	Pluripotent and highly proliferative; Patient‐specific for personalized medicine; Stable hepatic‐specific functions	Complex induction and culture; Limited functional maturation; Incomplete metabolic function
Hepatic‐derived cell lines	Stable phenotype and immortalized; Continuous proliferative capacity; Easy to handle	Metabolic limitations; Deficient transport protein expression; Low physiological relevance

**TABLE 2:** Origins and functions of non‐parenchymal cells.

The type of NPCs	Common sources	Functions
Liver sinusoidal endothelial cells [26]	Primary human liver tissue; iPSC‐derived, Hepatic cell lines (tumor cell line SK‐HEP‐1); Commercially available LSEC lines	Metabolic and physiological regulatory functions; Involved in immunity and inflammation; Substance exchange and clearance functions
Hepatic stellate cells [26, 27, 28]	Primary hepatic stellate cells; HSC‐T6 cell line; iPSC‐ derived HSCs	ECM secretion; Regulation of liver fibrosis; Vitamin A storage; Tissue regeneration and differentiation
Kupffer cells [26, 29]	Primary Kupffer cells; Immortalized or tumor‐ derived Kupffer cell lines; Commercially available KCs; iPSC‐derived KCs	Phagocytosis and clearance; Metabolic regulation; Immune surveillance and response

##### 2.1.1.1 Primary Human Hepatocytes (PHHs)

Primary human hepatocytes (PHHs) are considered the gold standard for constructing in vitro liver models due to their superior ability to simulate liver functions in a laboratory setting [15]. They exhibit excellent in vitro liver function simulation capabilities post‐isolation, including maintaining phase I and II enzyme activities crucial for drug metabolism, synthesizing albumin, bile acids, cholesterol, and other vital substances, as well as demonstrating transamination and glucose metabolism. However, these highly differentiated cells rapidly lose their specific functions when cultured in traditional two‐dimensional in vitro environments, evidenced by decreased expression and activity of cytochrome P450 enzymes (CYP450), rendering them unsuitable for extended in vitro culture and experimental research. Furthermore, the isolation of PHHs presents significant challenges due to limited donor availability and the technical complexity of the procedure [16]. Surprisingly, co‐culture of hepatocytes with non‐parenchymal cells (NPCs) is an effective method to maintain hepatocyte function in vitro [17]. This co‐culture mode can simulate the complex interactions between multiple cells under specific drug exposure, as well as the hepatotoxicity response mediated by the multicellular interactions and cell‐matrix interactions. Therefore, combining the co‐culture technology of PHHs and NPCs with LOC technology provides a new choice for in vitro drug screening and hepatotoxicity monitoring.

##### 2.1.1.2 Stem Cell Induces Hepatocytes (iPSC/HPCs/ASCs)

Stem cells possess attributes of pluripotency and proliferative capacity, establishing them as a promising model for various applications. Progenitor cells, derived from stem cells and yet immature, can be cultured within the specific microenvironments provided by organ‐on‐chips devices to facilitate their maturation. Hepatocytes induced from pluripotent stem cells, characterized by high proliferative ability and retention of pluripotent potential in culture, have emerged as a novel cell source. Researchers have successfully cultivated vascularized and functional human liver organoids from induced pluripotent stem cells by transplanting in vitro‐cultivated liver bud tissues (iPSC‐LBs). The iPSC‐LBs, when cultured in vitro, bear resemblance to in vivo liver buds. Through the formation of functional blood vessels, these iPSC‐LBs can progressively mature into human liver tissue. Tissues derived from iPSCs are capable of executing liver‐specific functions, such as albumin secretion and drug metabolism [18]. Hepatocytes differentiated from iPSCs exhibit lower levels of enzymatic activity and gene expression compared to normal liver tissues or hepatocytes obtained from human isolates. Nevertheless, hepatocytes induced from pluripotent stem cells can be employed to assess hepatotoxicity in acute treatments, to devise personalized medicine/treatment strategies by integrating patient‐specific iPSCs with LOC technology, and for chronic drug exposure and repeated‐dose studies, thereby addressing certain limitations associated with other cell lines [15].

##### 2.1.1.3 Hepatic‐Derived Cell Lines

To address the limitations of human primary hepatocytes, characterized hepatic‐derived cell lines such as HepG2, Huh7, and HepaRG have been employed in liver disease investigation, pharmaceutical screening, and cytotoxicity evaluations. Notably, these cells offer a stable phenotype, unlimited lifespan, and continuous proliferation within an in vitro culture environment, coupled with user‐friendly handling traits that facilitate laboratory operations. Nevertheless, when juxtaposed with human primary hepatocytes, these cell lines display diminished metabolic capabilities. For instance, the enzymatic activity of CYP450, integral to Phase I drug metabolism, is significantly lower than in human primary hepatocytes. Additionally, the expression of specific transporter proteins like the sodium taurocholate co‐transporting polypeptide is either suboptimal or absent [19]. Nevertheless, the two‐dimensional (2D) culture of hepatic‐derived cell lines continues to serve as a critical tool in the initial phases of safety evaluations. In addition, 3D spheroid cultures of some kinds of LOC cell lines have been shown to have higher expression of liver‐specific functional genes than 2D cultures, and this finding also provides a new idea for the construction of LOC based on 3D hepatic‐derived cell line spheroids [20].

##### 2.1.1.4 Non‐Parenchymal Cells

The liver is primarily composed of parenchymal cells, but also contains a diverse array of non‐parenchymal cells (NPCs), such as LSECs, KCs, HSCs, and liver‐resident lymphocytes. These NPCs play indispensable roles in maintaining liver architecture and functional homeostasis (Table 2). In LOC technology, the incorporation of NPCs is critical not only for reconstructing complex intercellular networks but also for mimicking the dynamic microenvironment of the liver, thereby enhancing the physiological and pathological relevance of in vitro models [21, 22, 23]. Importantly, NPCs interact closely with parenchymal cells to co‐regulate liver function [22, 24]. For example, LSECs secrete cytokines that influence hepatocyte polarity and metabolic activity; KCs sense pathogen‐associated molecular patterns (PAMPs) and initiate immune responses, while also releasing inflammatory mediators that affect hepatocyte behavior; HSCs, in their quiescent state, support hepatocyte growth, but upon activation, they secrete cytokines and extracellular matrix components that drive liver fibrosis. Conversely, hepatocytes can modulate NPCs' behavior through the release of exosomes, signalling molecules, and metabolic byproducts. This bidirectional communication between parenchymal and non‐parenchymal cells is essential for maintaining liver homeostasis, responding to injury, and orchestrating regeneration [23, 25].

Although early LOC platforms mainly focused on the culture and function of parenchymal cells, recent studies have increasingly highlighted the pivotal roles of NPCs in regulating tissue homeostasis, intercellular signaling, immune responses, angiogenesis, and drug‐induced toxicity. Therefore, the integration of multiple types of NPCs into LOC systems significantly enhances their physiological relevance and predictive capability, particularly for modelling liver diseases such as hepatitis, non‐alcoholic fatty liver disease, and liver fibrosis, as well as for applications in drug screening and toxicological evaluation [21].

#### 2.1.2 LOC Manufacturing‐Substrate Materials

**FIGURE 2:**
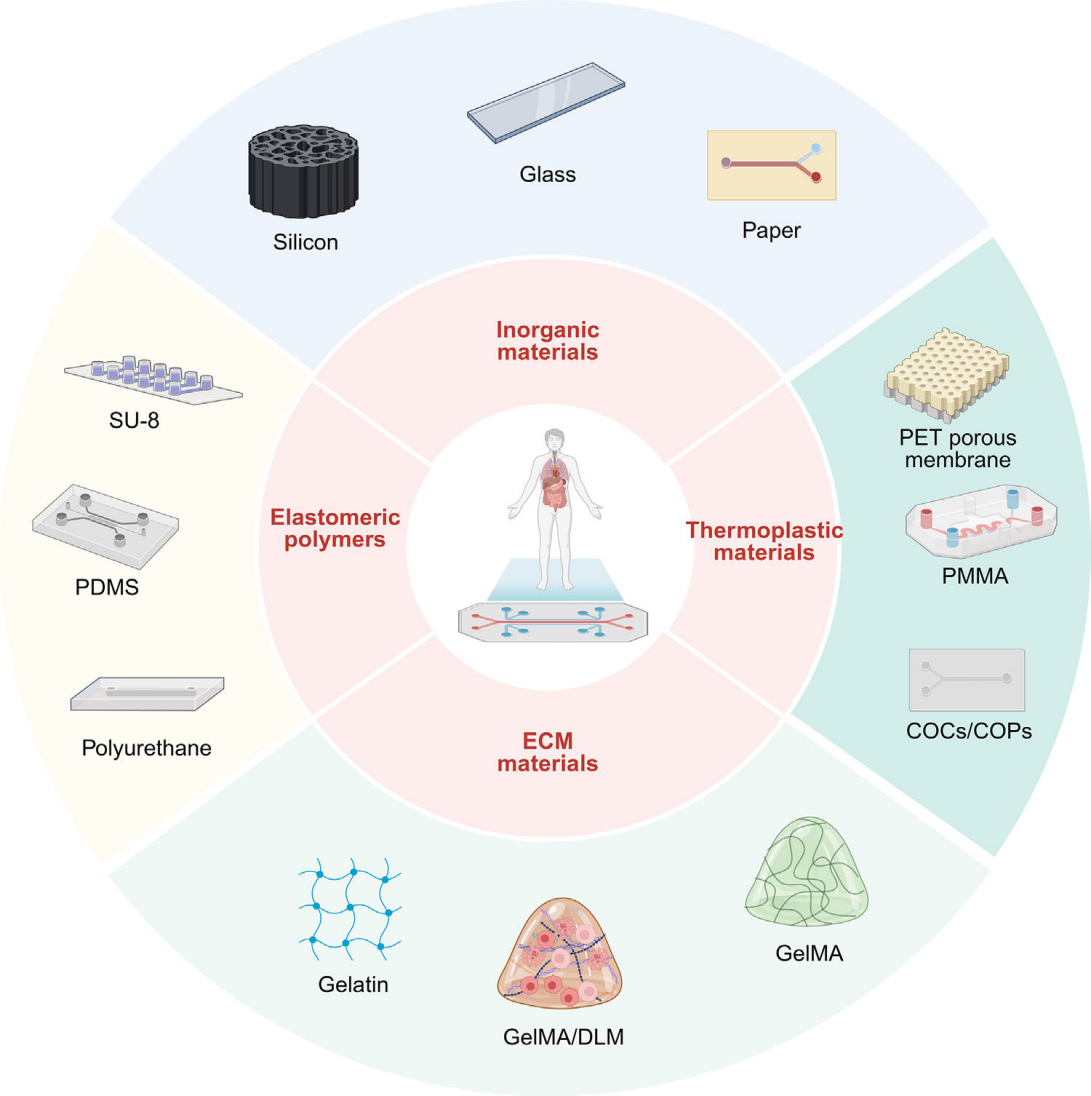
Classification of materials applied in LOC devices (Created in BioRender).

The core of OoC technology is microfluidics, which enables precise control of microscale fluid flow to replicate the complex hemodynamic environment and material exchange processes that occur in vivo. To construct the intricate 3D structures of OoC, including microchannels, microchambers, and multilayered interfaces, a wide range of materials has been introduced and continuously optimized. These materials must combine excellent processability, which allows for high‐resolution micro and nanostructure fabrication, with key properties such as biocompatibility, optical transparency, and chemical stability, ensuring their suitability for biological applications. These materials have been systematically summarized in Table 3. The integration of inorganic substances, elastomeric polymers, thermoplastic polymers, and ECM materials has been pivotal in advancing chip design (Figure 2).

**Inorganic materials**: In the field of OoC technology, silicon and glass are widely used as the main inorganic materials. The first generation of microscale cell culture systems, which mimic the complexity of human organs, was primarily constructed using silicon and glass [30]. These materials were highly utilized in the early applications of microfluidics because they were suitable for microfabrication. Glass, with its excellent optical transparency, is an ideal material for real‐time monitoring of cellular activities within devices. This clarity also minimizes the potential absorption of hydrophobic molecules and biomolecules, ensuring a more controlled experimental environment. Woolley et al. microfabricated capillary electrophoresis array chips on glass substrates using photolithography and chemical etching techniques and successfully achieved ultrafast DNA fragment separation. The glass microchannels provided better performance than other material chips due to the high thermal conductivity and stable electroosmotic mobility of their surfaces [31]. However, the non‐permeable nature of glass poses challenges for long‐term cell studies, as it limits the possibility of conducting experiments in enclosed glass microchannels and chambers. On the other hand, silicon offers a unique set of benefits, including resistance to organic solvents, ease of metal deposition, high thermal conductivity, and stable electroosmotic mobility. These characteristics make silicon an attractive choice for constructing OoC devices. Sin et al. designed a “lung‐liver‐other” three‐chamber cell culture device on a silicon chip. They leveraged the ease with which silicon materials integrate with other sensors or detection elements, along with their high conductivity, to develop an in vitro physiological pharmacokinetic (PBPK) model and successfully integrate dissolved oxygen sensors. The device provides a powerful tool for studying exogenous chemicals' toxicological and pharmacological properties [7]. Nevertheless, the manufacturing process of silicon and glass chips usually involves standard photolithography techniques, which can be both expensive and time‐consuming. These materials often require processing in high temperature, high pressure, and ultra‐clean environments, potentially complicating the procedure. Silicon is particularly costly, opaque to visible and ultraviolet radiation, and rigid, making the implementation of control units (such as pumps and valves) more challenging [32]. Moreover, both silicon and glass are impermeable and not suitable for studying living cells. Therefore, despite their use in building the first generation of microscale cell culture systems, over time, they have gradually been replaced by superior materials due to the more evident advantages of polymer materials.

Paper, as a porous matrix material made of cellulose, is characterized by its high flexibility, good biocompatibility, and low cost. During the preparation of paper‐based microfluidic devices, the aqueous solution on the paper matrix can be precisely guided through the hydrophilic region by modifying some regions hydrophobically, thus extending the patterned paper‐based microfluidic channeling approach [33]. Meanwhile, paper is used as a microfluidic device matrix for biochemical analysis and analyte detection. Detection methods include colorimetric, luminescent, and electrochemical detection [34]. Ebrahimi et al. reviewed the application of electrochemical microfluidic paper‐based analytical devices (µPADs) for cancer biomarker detection. Among them, in three‐dimensional µPADs, 3D spatial flow of fluids can be realized by stacking and folding paper‐based materials, which demonstrate the potential of paper‐based microfluidic devices for analyte detection applications [35]. Xie et al. developed modular 3D paper‐based microfluidic chips using projection‐based 3D printing (PBP). They assembled these 3D chips from 2D layers and combined them with extrusion‐based bioprinting. By printing 3D hydrogel structures directly onto the chips, they created a multi‐organ microfluidic chip system. This work demonstrates the potential applications of paper‐based microfluidics in biomedical engineering [36]. However, paper materials also have drawbacks; the fabric matrix in the paper base blocks internal signal transduction and dilutes the sample; hydrophobically modified channels do not confine low‐surface tension liquids well; and semi‐enclosed paper‐based microfluidic channels cause evaporation of liquids. However, paper‐based microfluidic devices still have good prospects in low‐cost portable commercial detection.

**Elastomeric polymers**: Elastomeric polymers have already made a significant impact in the field of OoC and flexible microfluidic devices due to their excellent elasticity and stability. They can be easily stretched and compressed by external forces and can be restored to their original state after the external forces disappear. The field of bio‐microfluidics experienced rapid growth in the late 1990s due to the introduction of polydimethylsiloxane (PDMS) [37]. PDMS offers several advantages, such as chemical stability, optical transparency, resilience, biocompatibility, and cost‐effectiveness. It can also be surface‐modified and used for prototyping new devices through soft lithography and microform technology, achieving the desired functionality [38]. For example, Ma et al. designed a reversible concave microporous PDMS‐membrane‐PDMS sandwich multilayer chip for perfusion culture of 3D cell spheroids, enabling maintenance of cell activity, morphology, improved cell polarization, metabolism, and liver‐specific functions [39]. Weng et al. developed a LOC using micropatterned PDMS to culture primary human hepatocytes, mimicking the portal vein flow without the need for exogenous scaffolds [40]. However, PDMS also has some drawbacks. Firstly, the PDMS polymer can absorb hydrophobic small molecules, and as a material substrate, it can have an impact on the solubility of soluble factors in the culture medium, interfering with functions such as intercellular signal transduction, and also misleading qualitative and quantitative analyses of small molecules secreted by the cells and thus affecting the accuracy of experimental results [41]. Secondly, uncured oligomers leached from the PDMS polymers, uncrosslinked oligomers can diffuse into the solution of the chip system, and it was found that this type of oligomers can penetrate the membranes of the cells, affecting the cellular activity [42]. Thirdly, the unstable surface properties of PDMS can introduce uncertain dynamics to cell culture devices. In addition, PDMS is incompatible with some organic solvents, leading to swelling. This leads to changes in the size and surface properties of microchannels and microchambers made of PDMS [43]. Surface modification of the PDMS or the use of alternative materials provides a solution, and some elastomers with molecular uptake resistance, such as polyurethanes, tetrafluoroethylene propylene, SU‐8 Polymers, etc., have become alternative materials for PDMS. In summary, PDMS has become a widely used material in OoC applications due to its unique properties. However, its limitations and potential drawbacks must also be considered when selecting it for use.

PDMS, with its tunable stiffness and structural properties, has been shown to significantly regulate cellular behavior, and can be customized to match the mechanical characteristics of specific tissues, thereby better recapitulating the physiological microenvironment [44]. The surface of PDMS can be microstructured by soft lithography to act as a patterning cue for cell culture and to guide cellular behavior and function. It supports surface modification by hydrophilic modification and coupling agent coatings grafted with ECM proteins, biofunctionalized alginate, and gelatin, which allow for long‐term cell adhesion and growth [45, 46, 47, 48].

**Thermoplastic synthetic materials**: Thermoplastic synthetic materials can be reshaped after deformation, such as poly (methyl methacrylate) (PMMA), poly (ethylene terephthalate) (PET), cyclic olefin polymers (COPs), cyclic olefin copolymers (COCs), and polystyrene (PS). These materials are highly malleable, optically transparent, rigid, biocompatible, and low‐cost. They are also less susceptible to monomer leaching and hydrophobic small molecule uptake compared to PDMS [49]. Additionally, thermoplastic composites can be functionalized through dynamic coating and surface grafting techniques depending on specific requirements. PMMA is a typical thermoplastic synthetic material with good thermal stability and permeability compared to silicon and glass. Based on PMMA's good transparency, chemical stability, mechanical properties, and processing simplicity, Chen et al. developed a PMMA microfluidic chip with high precision and performance based on CO_2_‐laser micromachining and thermal bonding [50]. CO_2_ laser micromachining technology is characterized by high precision, high efficiency, and high flexibility. It enables precise processing of PMMA materials and fabrication of microstructures. By continuously optimizing the design, processing, and connection methods of microfluidic chips, more complex and accurate organ chips can be fabricated, which provides a new way for the fabrication of OoC. Polyethylene Terephthalate (PET) is a widely used thermoplastic polymer, and PET membranes have been used in many OoC and microfluidic cell culture platforms. It is a non‐degradable and transparent material, and its cell adhesion can be increased by plasma treatment. In LOC applications such as drug screening, continuous perfusion conditions and a steady flow of cells for culture are essential. Hegde constructed a dual‐chamber chip with two layers of PDMS separated by a porous PET membrane. The lower chamber is used to culture hepatocytes, and the upper chamber is perfused with fluid. The PET membrane serves as a barrier to isolate the collagen gel while allowing soluble factors to be exchanged across the membrane in the upper and lower layers [51]. COCs/COPs are amorphous thermoplastic copolymers, and COCs and COPs are highly resistant to acids, bases, and polar organic solvents, in addition to having low water absorption, good optical properties, and excellent biocompatibility, and have extremely low impurities compared to other thermoplastic materials [52]. Taking advantage of the fact that COPs do not absorb hydrophobic small molecules, Wen et al. established a model of non‐alcoholic fatty liver disease by using COPs to build a microphysiological system and introducing free fatty acids (FFAs) to induce HepaRG cell expression [53].

Thermoplastic synthetic materials exhibit numerous advantages in OoC fabrication, such as excellent chemical stability, high throughput and consistency, good sealability, ease of molding, and a wide range of material options with modifiable surface properties. These characteristics make them suitable for mass production and optical detection applications. However, these materials also present certain limitations, including poor gas permeability, compatibility issues with organic solvents, poor cell adhesion, potential small molecule penetration issues, and relatively higher costs and thermal expansion problems. Particularly, additional processing may be required in terms of microscale precision and biocompatibility. For rapid prototyping, novel materials have been developed, such as photocurable liquid PS prepolymers compatible with soft lithography. This is expected to bridge the gap between “microfluidic prototyping,” “industrial microfluidics,” and “applied microfluidics” communities [54]. Therefore, thermoplastic synthetic materials provide a reliable and scalable alternative in OoC fabrication, yet they have their limitations, especially regarding microscale and biocompatibility, which may require additional processing and consideration. Researchers must weigh these pros and cons according to specific application requirements and budget when selecting materials (Table 3).

**TABLE 3:** Advantages and limitations of the substrate materials in the LOC.

Materials type	Materials	Advantages	Limitations
Inorganic materials	Silicon	Resistant to solvents; Thermally conductive & stable flow; Sensor integration capable; Multi‐organ & pharmacokinetic (PBPK) compatible	Light‐blocking; High‐cost fabrication; Rigid & non‐permeable; Integration limited
	Glass	Optically transparent; Thermally conductive & stable flow; Low adsorption; Microfabrication compatible	Gas impermeability; Fabrication‐intensive; Brittle & rigid
	Paper	Low‐cost material; Good compatibility; Flexible and foldable; Supports 3D microfluidics; Supports capillary flow; Detection compatible	Poor confinement of low‐tension liquids; Fibers may block signals; Evaporation affects accuracy
Elastomeric polymers	PDMS	Excellent elasticity; Chemically stable & biocompatible; Low cost & easy processing; Tunable stiffness & surface	Absorbs small molecules; Leaching of oligomers; Unstable surface properties; Swelling in solvents
Thermoplastic synthetic materials	PMMA	Thermally stable & gas permeable; Transparent & robust; Easy processing; Low cost and functionalizable	Rigid, poor elasticity; Swelling risk; Poor hydrophilicity
	PET	Structurally stable and non‐degradable; Enhanced cell adhesion; Porous membrane compatible	Less machinable; Rigid, not flexible; Narrow temp range
	COPs/COCs	Optically clear & biocompatible; No hydrophobic uptake; Chemically resistant; Low water absorption	Higher material cost; Complex processing; Limited functionalization

#### 2.1.3 LOC Manufacturing‐ECM Bionic

The extracellular matrix (ECM) serves not only as a structural scaffold for cells but also as a dynamic, bioactive network that orchestrates key physiological processes, including cell proliferation, differentiation, migration, and apoptosis [55]. In native tissues, the ECM plays a central role in regulating cellular behavior and tissue functionality through its complex biochemical composition, mechanical properties, and spatial organization. As a cutting‐edge in vitro model that integrates microfluidics, bioengineering, and cell biology, OoC technology aims to replicate the physiological microenvironment and functional characteristics of human organs. Within this context, the reconstruction and biomimetic design of ECM components are pivotal for achieving accurate tissue‐level functional emulation and long‐term biological relevance in OoC systems (Figure 2).

Hydrogel, as a polymeric material, stands out due to its inherent ability to mimic the key structural features of natural ECM with good biocompatibility and controllability, such as elasticity, porosity, degradability, and permeability, and is therefore used for the simulation of natural ECM components in chips. Natural hydrogels such as collagen, gelatin, chitosan, fibronectin, and alginate have excellent biocompatibility and physicochemical properties. The collagen family constitutes the most abundant protein within the ECM, and its fibrous structure contributes significantly to the formation of the ECM. As a major component of connective tissues, collagen provides essential support for the stability of cells, organs, and tissues [56]. Due to its excellent biocompatibility and favorable physicochemical properties, collagen has also been utilized as a chip material. Toh's group designed a liver chip featuring multiple hepatic plate structures for in vitro drug hepatotoxicity assays. They created a 3D microenvironment mimicking the ECM by perfusing positively charged collagen and negatively charged acrylate‐based copolymers into the cell culture chamber. The results demonstrated that this 3D microenvironment enhanced cell‐cell and cell‐matrix interactions and could maintain the activity and function of hepatocytes over an extended period [57]. Gelatin, a natural polymer derived from the controlled degradation of collagen, exhibits excellent biocompatibility and degradability due to its diverse biological origins [58]. Kazuki Sasaki et al. employed fibronectin‐coated gelatin nanomembranes to sequentially layer human primary hepatocytes, human umbilical vein endothelial cells, and human dermal fibroblasts, thereby constructing a homogeneous, dense, and vascularized liver tissue [59]. This approach significantly enhanced albumin production and CYP450 enzyme activity in hepatocytes.

Natural hydrogels, however, have certain limitations, including relatively poor mechanical properties, limited long‐term stability, and batch‐to‐batch variability. Gelatin methacrylate (GelMA) is derived from modified gelatin and retains essential ECM properties, such as the presence of arginine‐glycine‐aspartic acid sequences that promote cell attachment and matrix metalloproteinase‐responsive peptide motifs, facilitating cell adhesion and proliferation within GelMA scaffolds. Also, GelMA exhibits photocrosslinking capabilities, allowing the preparation of hydrogel scaffolds with tunable mechanical properties through varying crosslinking methods.

**FIGURE 3:**
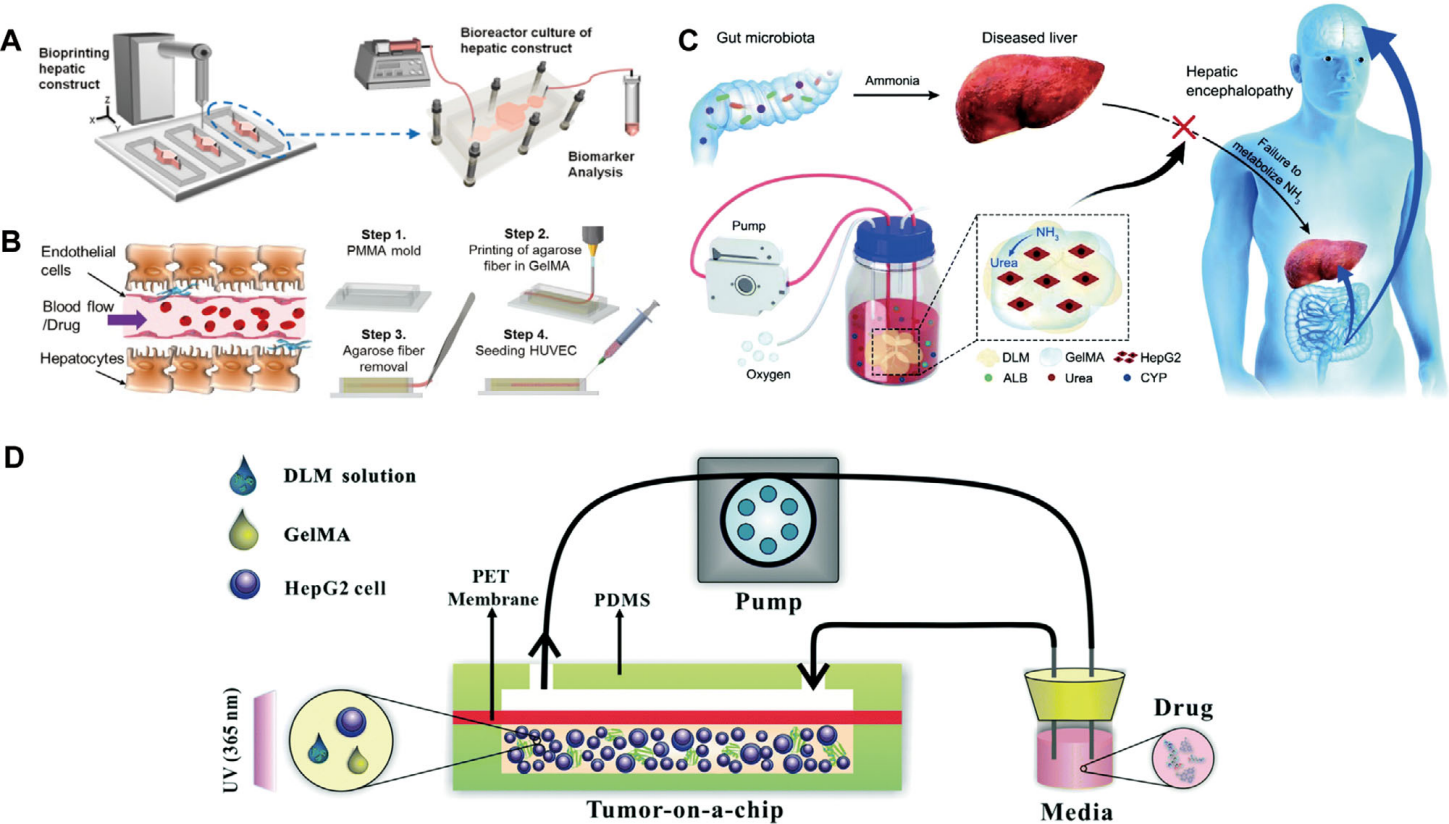
Hydrogel‐based biomimetic ECM OoC. (A) The principle diagram of an LOC platform integrated with a bioprinter for printing GelMA hydrogel as ECM. Reproduced with permission [60]. Copyright 2016, IOP Publishing Ltd. (B) Hollow microchannels were created in GelMA hydrogels encapsulated with HepG2/C3A cells using sacrificial bioprinting to simulate the matrix and liver blood flow. Reproduced with permission [61]. Copyright 2017, American Institute of Physics. (C) Functional characterization of HepG2 cells under DLM/GelMA combination as ECM. Reproduced with permission [63]. Copyright 2020, Royal Society of Chemistry. (D) Schematic of HepG2‐laden decellularized liver matrix with gelatin methacryloyl (DLM‐GelMA) in a microfluidic device. Reproduced with permission [64]. Copyright 2018, Royal Society of Chemistry.

GelMA has significantly advanced the field of LOC by facilitating the construction of hepatic physiological environments and aiding in drug screening, clinical diagnosis, and tissue regeneration in vitro. Nupura S Bhise utilized GelMA as a bio‐ink to print HepG2/C3A spheroids, which were cultured for 30 days via perfusion in a bioreactor (Figure 3A). These liver spheroids exhibited excellent cell viability and liver‐specific functions, including the secretion of albumin, α‐1 antitrypsin, transferrin, and ceruloplasmin. The platform mimics liver function by encapsulating 3D human‐derived hepatocyte spheroids and is capable of achieving long‐term stable maintenance of hepatocyte function, demonstrating the platform's potential in drug toxicity assessment and providing a more accurate in vitro model for drug development [60]. Massa et al. created a hollow microtubular channel and encapsulated HepG2/C3A cells within GelMA aggregates, forming vascularized tissue constructs with high cell viability and expression (Figure 3B) [61]. While GelMA replicates the basic mechanical characteristics of the extracellular matrix, it lacks endogenous growth factors and exhibits limited biomimicry of native tissue microenvironments. In contrast, decellularized extracellular matrix (dECM) retains essential tissue‐specific biochemical and structural cues, providing a bioactive scaffold that facilitates cellular adhesion, migration, proliferation, and differentiation, thereby enhancing tissue‐specific regeneration and functional restoration [62]. Wu et al. developed a novel artificial liver system by integrating GelMA and hepatocytes into a decellularized liver matrix (DLM). The GelMA/DLM combination enhances hepatocyte‐specific functions while providing natural growth factors for cell growth and biomechanical support to maintain the liver's natural structure (Figure 3C) [63]. To better replicate the natural tumor microenvironment, Lu et al. designed a microfluidic 3D dynamic cell culture system based on GelMA/DLM (Figure 3D). This bionic LOC with integrated DLM demonstrates improved ability to maintain cell viability and enhance hepatocyte function under flow conditions, offering a more accurate tumor microenvironment compared to GelMA alone [64]. In order to reproduce the key physicochemical cues of the native ECM, encapsulation of cells in other synthetic hydrogels (e.g., polyethylene glycol diacrylate, PEGDA) and natural biopolymer networks (e.g., agarose) has also been extensively explored as a basic strategy for engineering biomimetic microenvironments [65, 66]. Synthetic hydrogels have emerged as pivotal materials for constructing biomimetic microenvironments in LOC platforms; future advancements will focus on developing multifunctional hybrid systems that integrate bioactive factors to achieve precise physiological replication and enhanced function.

### 2.2 Classification and Construction Strategies of the LOC

**FIGURE 4:**
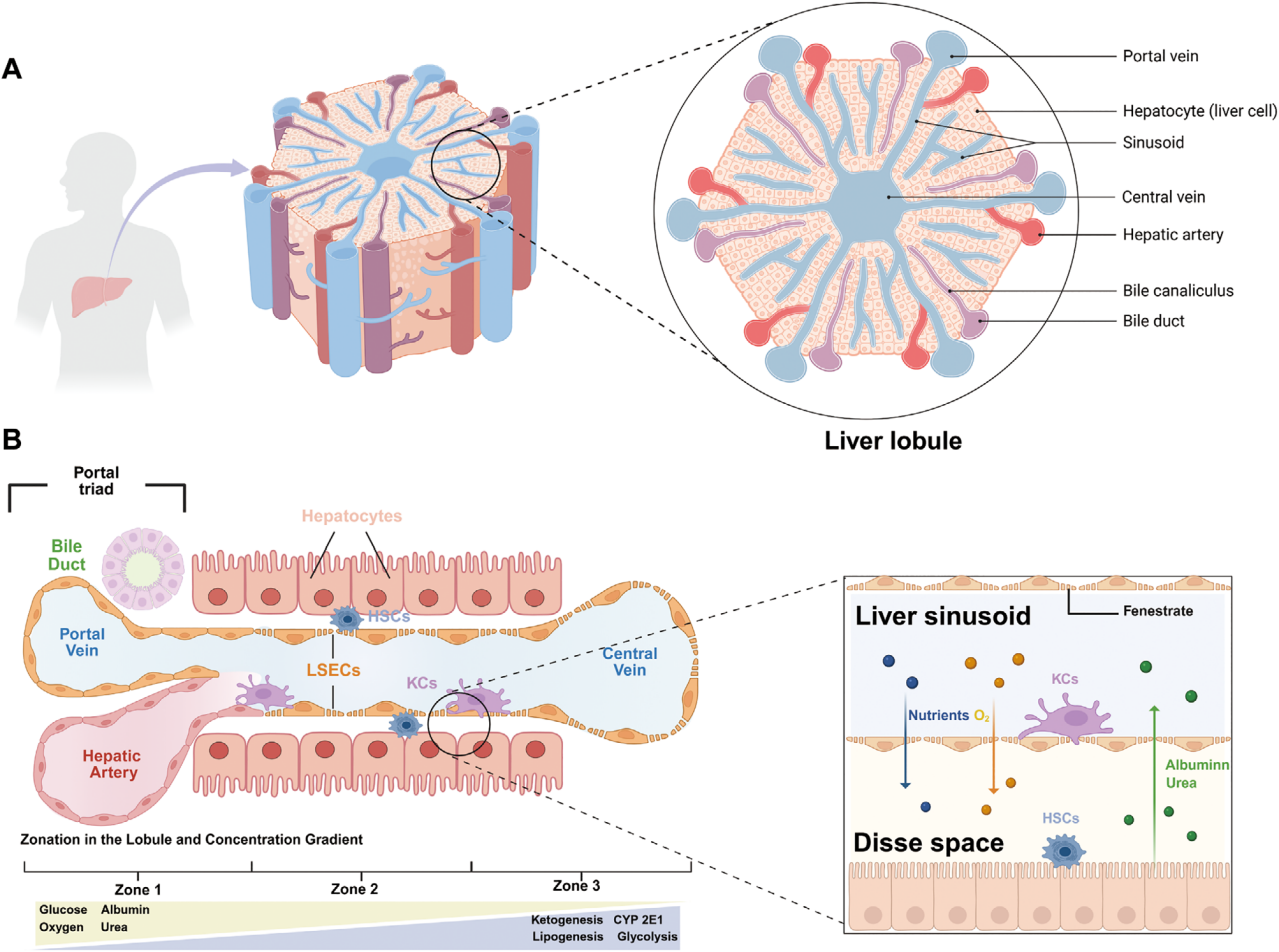
Overview of liver physiology. (A) The liver lobule is the basic structural and functional unit of the liver with a hexagonal shape. (B) Cellular composition, structural zonation, and material concentration gradients in the liver sinusoid (created in BioRender).

The liver, as the central organ for metabolism, detoxification, and biosynthesis, relies heavily on its intricate microarchitecture [1]. The liver lobule is the fundamental structural and functional unit of the liver. It has a roughly hexagonal shape with a central vein at the core and portal triads (comprising the portal vein, hepatic artery, and bile duct) at the corners. Hepatocytes are arranged in radial cords extending from the portal areas toward the central vein. Blood flows from the portal vein and hepatic artery into the liver sinusoids, traverses through the lobule, and drains into the central vein—creating a directional flow axis from the portal triad to the central vein. In contrast, bile produced by hepatocytes flows in the opposite direction toward the bile duct, establishing a unique countercurrent system (Figure 4A). Within this architecture, liver sinusoids serve as specialized microvascular channels that facilitate efficient exchange between the blood and hepatocytes. These sinusoids are lined with fenestrated endothelial cells lacking a basement membrane, enhancing permeability. They are also populated with various NPCs, including KCs and HSCs, which play vital roles in immune surveillance, inflammation regulation, and liver homeostasis. While the hepatic lobule represents the macroscopic organization and metabolic zonation, the sinusoid exemplifies the microscopic dynamics of cellular interactions and hemodynamic forces (Figure 4B). These two complementary structures have inspired the development of liver lobule chip and liver sinusoid chip platforms. The former mimics tissue architecture and metabolic zonation, while the latter focuses on microvascular flow and immune interactions.

#### 2.2.1 Liver Lobule Chip

The liver lobule, the basic functional unit of the liver, consists of radially arranged hepatocyte plates around a central vein and peripheral portal triads, forming a zonated structure that regulates metabolism and drug response. Liver lobule chips are widely used to replicate this architecture for studying liver function, disease modeling, and drug metabolism (Figure 4A).

**FIGURE 5:**
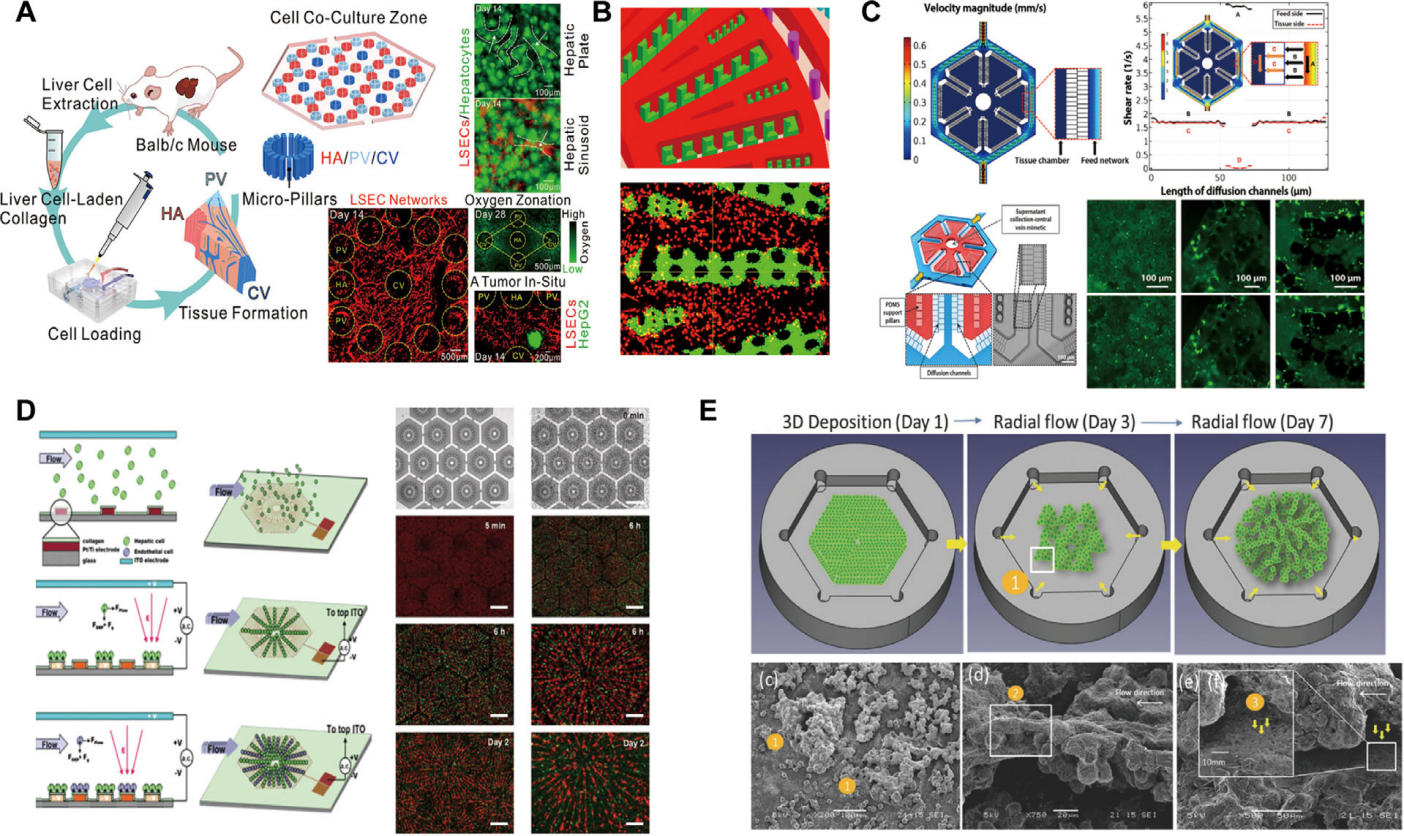
Liver lobule chip. (A) The LLC and construction of liver lobules with self‐assembled perfusable hepatic sinusoid networks. Reproduced with permission [67]. Copyright 2021, American Chemical Society. (B) 3D configuration of the biomimetic microfluidic device and morphological demonstration of the 3D liver lobule‐like microtissue. Reproduced with permission [68]. Copyright 2016, American Chemical Society. (C) In the VLSLL device, the simulation of flow velocity and shear rate facilitated the formation of three‐dimensional tissue‐like structures and bile duct networks. Reproduced with permission [69]. Copyright 2017, IOP Publishing Ltd. (D) DEP‐based heterogeneous lobule‐mimetic cell patterning and integration of HepG2 cells and HUVECs via DEP. Reproduced with permission [70]. Copyright 2013, Royal Society of Chemistry. (E) Morphogenesis of organotypic architecture with sinusoid wall‐like morphology and a fenestrated window‐like nanostructure under simulated three‐dimensional liver lobule blood flow perfusion, achieving the biomimetic reconstruction of the liver lobule tissue hierarchy. Reproduced with permission [40]. Copyright 2017, Wiley‐Blackwell.

In the realm of microengineering, Ya et al. pioneered a sophisticated liver lobule chip (LLC) featuring self‐assembled, perfusable hepatic sinusoid networks, addressing the formidable challenge of replicating in vitro the intricate vasculature inherent to liver tissue (Figure 5A) [67]. This achievement was realized by designing an innovative oxygen concentration regulating chip (ORC) that mimicked the in vivo dual‐vessel blood flow supply model, which reproduced the complex oxygen gradient environment inside liver tissues by connecting an ORC module to the portal veins (PV) and hepatic arteries (HA) inlets of the chip, and setting the dissolved oxygen concentrations of 4.2 mg L^−1^ and 1.8 mg L^−1^ for the HA and PV, respectively. By ingeniously utilizing micropillar arrays to compel the culture medium to flow through the gaps between the pillars at a controlled velocity, the low‐flow‐resistance medium disrupts weak collagen coagulation and facilitates the formation of flow pathways. This ultimately provides LSECs with opportunities for reorganization, thereby orchestrating the directional assembly of hepatic sinusoids. As a result, a radial network is established, culminating in the construction of a 3D liver lobule architecture that encompasses not only sinusoidal vasculature but also hepatocyte cords and biliary structures. Departing from conventional models that simplify vascularization through a single portal vein and hepatic artery representation, the researchers introduced a dual‐vessel supply system, capturing the nuanced differences in arterial and venous oxygen content observed in real‐life scenarios. This breakthrough led to the creation of a scaffold‐free, self‐assembling tissue interface, complete with functional hepatic sinusoids, marking a significant advancement in the development of biomimetic liver models for research and potential therapeutic applications.

Ma et al. designed a microfluidic platform to create patterned cell cultures and spatial arrangements by combining radial micropatterning with a pneumatic microvalve system [68]. The device was constructed to mimic liver lobule tissue, with a hexagonal culture region equipped with a double‐sized microcolumn array, which enabled stable immobilization of HepG2 cells within a collagen hydrogel within the culture chamber, and induced the cells to align along the columnar structure to form a “hepatic cord‐like” network. The control layer integrates an inner valve and an outer valve. During the cell loading phase, the inner valve drives the cell‐collagen hydrogel mixture to precisely position in the gap of the microcolumns by applying a pressure of about 18 psi, thus rapidly realizing the patterned arrangement of cells. After gelation, the valve could switch states to assist the loading of immortal human aortic endothelial cell lines (HAECs), which were uniformly distributed within the pre‐determined channels formed by the collagen hydrogel, ultimately constructing a bionic hepatic sinusoidal vessel‐like network. The synergistic action of the micropillars and air valves enables the automation and high‐precision manipulation of the whole process from cell localization and patterning to 3D tissue construction. Through this design, HepG2 cells and HAECs were able to form highly mimetic hepatic cord and sinusoidal structures, which not only enhanced cell survival and metabolic function, but also enhanced the sensitivity of drug‐induced and inhibited responses, providing a reliable platform for hepatotoxicity and drug‐drug interaction studies (Figure 5B).

Amin A Banaeiyan et al. developed a high‐throughput large‐scale liver‐lobule (VLSLL)‐on‐a‐chip device, inspired by human blood flow mechanics [69]. This LOC features an integrated hexagonal tissue culture chamber layer resembling the liver lobule, complete with material inlet and outlet layers. The hepatocyte culture region is separated from the surrounding feed network channels by a double‐walled arrangement of microfluidic channels (separating chamber walls). The design of the separating chamber walls both protects the cells from convective shear forces and relies on diffusion‐convection effects to achieve nutrient support for the cells in the culture zone. Each culture chamber includes an outlet channel that mimics the central vein's physiological function through fluid circulation between upper and lower layers. Individual PDMS columns within the culture zone provide mechanical support for the cells and allow them to attach in a radial arrangement to form tissue‐like structures. The culture of HepG2 cells and hiPSC‐derived hepatocytes revealed maintained cell morphology and physiological functions, along with the formation of three‐dimensional tissue‐like structures and bile duct networks (Figure 5C).

Ho et al. proposed a chip design that mimics the structure of the liver lobule, the core of which lies in the precise patterning manipulation of HepG2 and HUVECs through microstructured electrode arrays by using enhanced electric field‐induced dielectrophoresis (DEP) technology (Figure 5D) [70]. The chip adopts a dual‐layer electrode design, controlling the electric field distribution in the inner and outer rings respectively, corresponding to the localization of different types of cells, thus simulating the radial arrangement of hepatocytes and the endothelial cells surrounding them in the real liver lobules. The specific electrode shape is a star‐shaped radial configuration, which attracts the cells to the maximum electric field gradient with the help of positive dielectrophoretic force, and realizes the orderly arrangement of heterogeneous cells through a two‐step process of loading and fixing the hepatocytes first, and then loading the endothelial cells. The chip was fabricated using micromachining technology, including titanium‐platinum metal electrode deposition, photolithographic pattern transfer, ITO glass encapsulation and other steps to construct a microfluidic chamber with high conductivity and biocompatibility. The design not only spatially realizes single‐cell resolution arrangement, but also maintains more than 95% of cell activity and shows a significant increase in the activity of liver enzyme CYP450‐1A1, demonstrating that this mimic liver lobule chip has made breakthroughs in both structural reconfiguration and functional mimicry.

Inspired by lattice growth mechanisms, Weng et al. employed microengineering techniques to manipulate primary liver cells (PLCs) into liver lobule structures [40]. They initially deposited primary hepatocytes onto collagen‐coated PDMS membranes to create growth templates and sealed them after overnight incubation to form a culture chamber with hydrophilic steering gears for vertical anchoring of PLCs. The culture chamber was then assembled with a reservoir to establish fluid circulation. A pump facilitated medium flow between the reservoir and blood vessel‐mimicking microchannels, entering through a hexagonal apex inlet and exiting via the central outlet, simulating liver lobule blood flow. Under three‐dimensional perfusion, PLCs transformed from round clusters into tissue‐like structures featuring sinusoidal walls and open window‐like formations (Figure 5E). Consequently, they developed a LOC system that recreates portal vein to central vein blood flow without needing an exogenous scaffold, achieving a bionic reconstruction of the liver lobule's tissue hierarchy by incorporating physiologically relevant extracellular matrices through primary hepatic stellate cells (HSCs) assembly.

#### 2.2.2 Liver Sinusoid Chip

The liver sinusoid, the smallest vascular structure within the liver, is a capillary channel between hepatocyte plates lined with fenestrated endothelial cells, facilitating efficient exchange of substances between blood and hepatocytes. Liver sinusoid chips are often used in biomedical research to better understand the physiological functions, pathological changes, and drug metabolism processes in the liver (Figure 4B).

**FIGURE 6:**
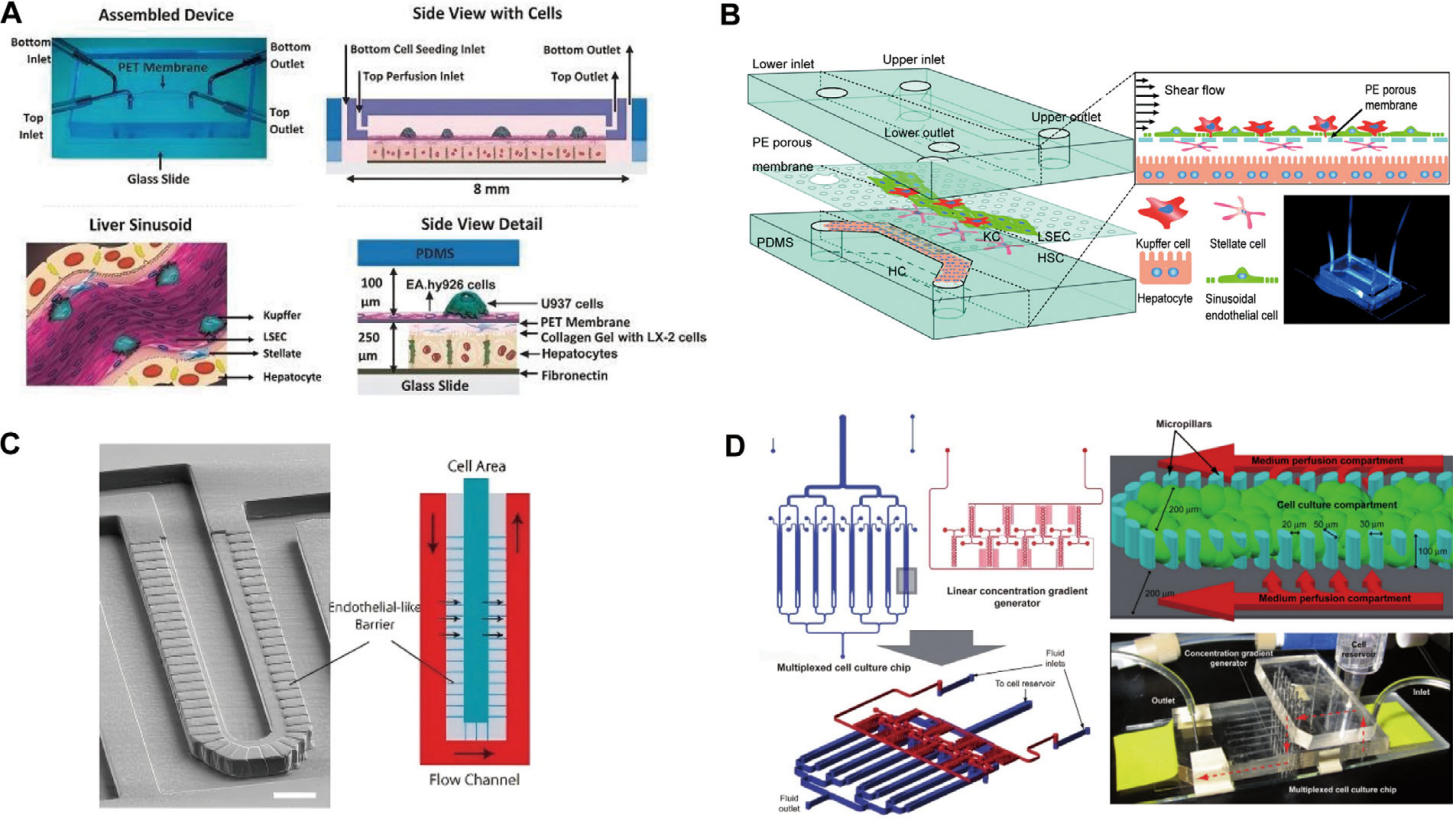
Liver sinusoid chip. (A). Schematic diagram for fabrication of a two‐chambered liver sinusoidal microfluidic chip. Reproduced with permission [71]. Copyright 2016, Wiley‐VCH Verlag. (B) Schematic of the in vitro 3D liver sinusoid chip. Reproduced with permission [72]. Copyright 2017, Royal Society of Chemistry. (C) Microfluidic endothelial‐like barrier properties simulating the hepatic sinusoidal structure. Reproduced with permission [73]. Copyright 2007, Wiley‐VCH Verlag. (D) Microfluidic design and assembly of the linear concentration gradient generator and multiplexed cell culture chip to construct the liver sinusoid chip. Reproduced with permission [57]. Copyright 2009, Royal Society of Chemistry.

Prodanov et al. developed an advanced microfluidic chip to better mimic the liver sinusoid architecture and physiological milieu [71]. The device featured a dual‐chamber system separated by a PET porous membrane, enabling co‐culture of PHHs with non‐parenchymal cells under dynamic flow conditions (Figure 6A). PHHs were seeded in the lower chamber, while LX‐2 cells embedded in collagen gel were positioned between the hepatocytes and the PET membrane to emulate the Disse space. On the opposite side, EA.hy926 cells formed a monolayer, representing the endothelial lining of the sinusoids. The PET membrane acted as both a barrier preventing collagen infiltration and a conduit for efficient material exchange. U937 cells were cultured in the upper chamber to substitute for Kupffer cells (KCs). This flow‐based system significantly enhanced and stabilized the secretion of urea and albumin, as well as maintained consistent CYP3A4 activity, due to continuous nutrient supply and waste removal, compared to static cultures.

Du et al. created a microfluidic chip featuring a two‐channel design separated by a porous PE membrane to replicate the liver sinusoid structure and microenvironment. In the top chamber, liver sinusoidal endothelial cells (LSECs) were arranged on the upper side of the PE membrane, with Kupffer cells (KCs) sparsely distributed above them in a 2:1 ratio to mimic the liver sinusoid. Hepatic stellate cells (HSCs) were placed on the opposite side of the PE membrane to simulate the Disse space, also at a 2:1 ratio with LSECs. To emulate the liver's sinusoidal structure and microenvironment in vitro, Du et al. assembled Hepatocytes (HCs), LSECs, KCs, and HSCs in a layered, three‐dimensional configuration within a microfluidic chip (Figure 6B). This design aimed to replicate the cells in vivo arrangements around the liver sinusoid. Fluid shear stresses of 0.1 or 0.5 dyn cm^−2^ were applied in the upper chamber to mimic physiological blood flow. Subsequent analysis of hepatocyte‐specific secretion and cytokine interactions demonstrated the chip's ability to recapitulate liver sinusoid specific functions effectively [72].

Lee et al. constructed a microfluidic organ‐on‐a‐chip that mimics the microenvironment of the hepatic sinusoids, using a layer‐by‐layer modularized architecture, with a mechanical design that reproduces the functional structures of blood supply, material exchange, and cell arrangement in the hepatic lobules on a micro‐scale, and constructed three main functional units through micromachining technology: the main fluid pathway, the cell culture lumen, and the “microfluidic endothelial barrier” (Figure 6C). The endothelial barrier is designed for high fluid resistance, which ensures an almost shear‐free flow environment in the culture chamber and relies only on diffusion for nutrient and metabolic waste transfer, mimicking the natural low‐shear, diffusion‐dominated exchange of materials in the liver sinusoids. During chip loading, cells are injected through a single inlet and effectively confined within the culture chamber by a high‐resistance slit, resulting in a high‐density, tightly packed cellular structure that avoids shear damage and supports long‐term culture stability. Overall, the mechanical design of the organoids cleverly combines porous barriers, micro‐perfusion channels, and high‐density cell loading structures to highly restore the core functions of the liver sinusoids in terms of their physical geometry and mass transport properties, which provides a strong technological support for the construction of microscale multicellular bionic liver tissues [73].

Toh's group created an LOC with multiple liver sinusoid structures, comprising eight parallel single‐cell culture channels. Microcolumns in the cell culture zones effectively immobilize cells in the central region, creating a high‐density, three‐dimensional aggregation state while avoiding cell loss. The micropillar array not only serves as a physical barrier, but also maintains shear stability during perfusion of the culture solution to prevent cellular stress. Each channel is divided by micropillars into two side perfusion chambers and an intermediate cell culture area (Figure 6D). This configuration allows each channel to function as an independent drug concentration gradient reactor, with the medium and drug concentrations in the side perfusion chambers being individually controlled via a gradient generator for multi‐drug testing. The chip's design facilitates the measurement of hepatocyte activity and differentiation through albumin secretion and phase I and II metabolic activity assessments. Results indicated that this multi‐channel setup enhances cell growth, proliferation, functional maintenance, and metabolic activity [57].

#### 2.2.3 Liver Acinus Chip

**FIGURE 7:**
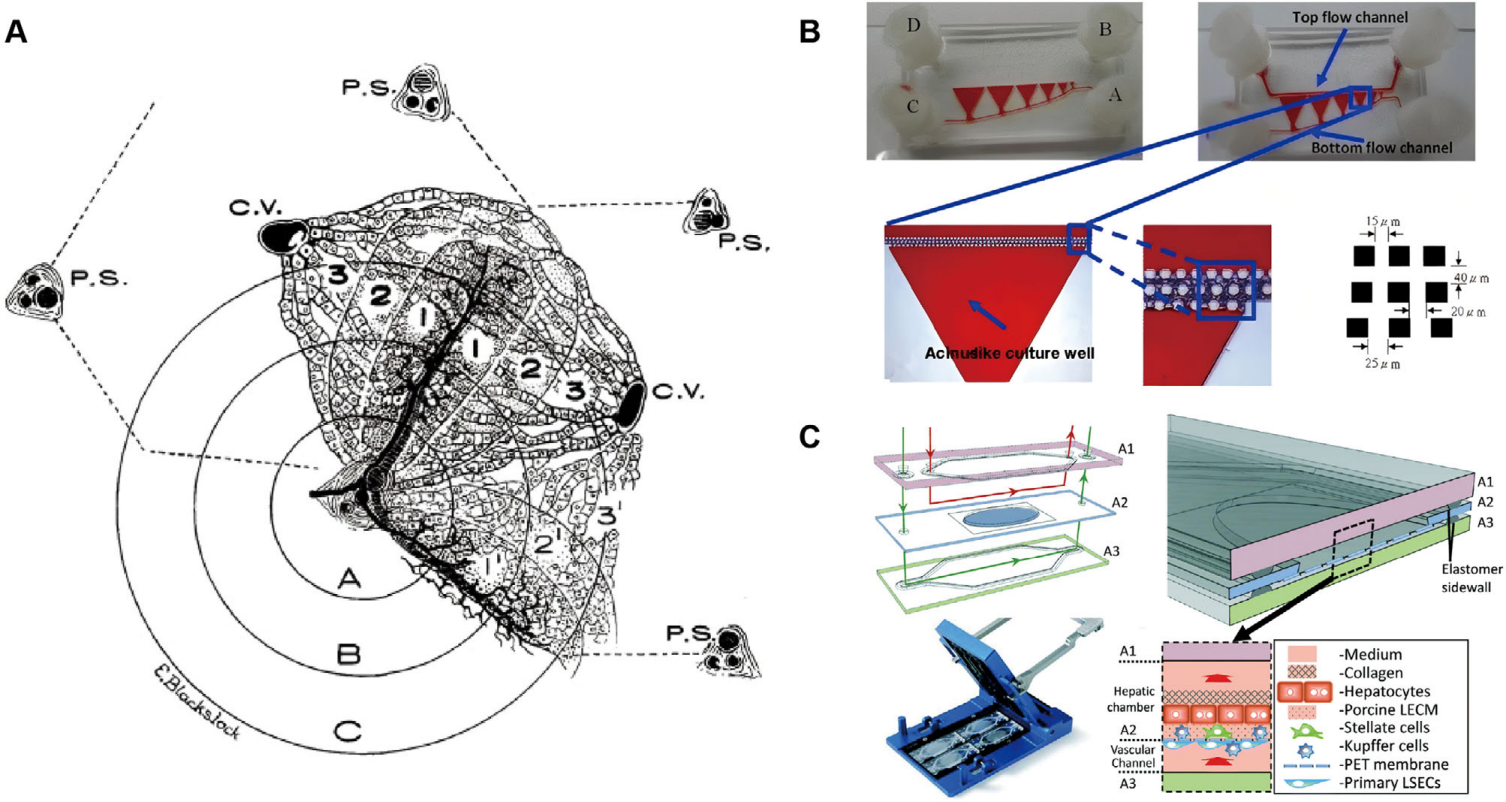
The structure of hepatic acinus and liver acinus OoC. (A) The structure of liver acinus. Reproduced with permission [74]. Copyright 1954, Wiley‐Liss, Inc. (B) Chip design for constructing concentration gradients and simulating the hepatic acinus physiological system with four medium inlets/outlets (ABCD) and 6 cell culture wells. Reproduced with permission [75]. Copyright 2013, Springer. (C) The vascularized liver acinus microphysiology system. Reproduced with permission [76]. Copyright 2018, Royal Society of Chemistry.

Rappaport's group introduced the concept of dividing liver lobules into smaller functional units called liver acinus in 1954 (Figure 7A) [74]. In this model, adjacent portal veins and central veins form a triangular structure through which blood flows from the portal vein and sinusoids into the central vein at the top of the triangle, also known as the “terminal portal vein.” The liver acinus can be divided into three regions along the direction of blood flow: proximal “region 1,” intermediate “region 2,” and distal “region 3.” These regions represent different metabolic zones with gradients in oxygen and glucose concentrations, albumin and urea production, and varying hepatotoxicity. Dividing the liver lobule into these smaller functional units enables a better understanding of liver physiopathological processes.

To replicate the liver concentration gradient microenvironment in vitro, Shih constructed a microfluidic liver acinus chip, which is a microstructural system with multiple functional zones precisely structured by soft lithography, aiming to highly mimic the zonal metabolic gradient of liver acinus and microcirculatory environment [75]. The chip consists of an upper and lower dual‐channel perfusion system with a triangular culture zone nested in the middle, and multiple rows of square microcolumns for shear stress buffering and fluid equalization. The chip is designed to mimic the oxygen and hormone distribution between different zones of the liver (Zones 1–3) by regulating the pressure difference and the geometric configuration of the semicircular channels to create a nonlinear, stable, and fast‐responding concentration gradient (Figure 7B). In addition, the chip supports a high‐density, gel‐free cell injection strategy, which ensures direct cell‐to‐cell contact and physiological functional expression; the combination of gravity and constant‐pressure drive ensures perfusion stability and significantly reduces metabolic toxicity and shear damage. This system allows rapid concentration gradient adjustment in the liver acinus while maintaining high‐density cell inoculation and contact in a gel‐free environment, inspiring subsequent chip designs that mimic the liver acinus structure.

Li et al. designed an optically transparent, glass‐based microchip model that mimics the physiological microenvironment of liver acinus, preventing hydrophobic small molecule uptake while enabling real‐time observation [76]. The device featured vascular microchannels and cell culture microchambers separated by porous membranes and inoculated with co‐cultures of PHHs, KCs, HSCs, and LSECs. A continuous oxygen gradient was created by controlling media flow rates in the vascular and liver channels to simulate the liver acinus environment (Figure 7C). They modeled and validated oxygen partitioning using oxygen‐sensitive and insensitive beads. Real‐time monitoring of cellular activities and interactions, such as immune function, cell migration, and biofactor secretion in response to inflammation, was facilitated by a fluorescent sensor device. This microchip model allows direct exploration of liver zonation roles in physiology/pathology, toxicology, and disease research, providing insights into individual and interactive relationships between parenchymal and nonparenchymal cells, inflammatory responses, and mechanisms of liver injury.

### 2.3 Functional Enhancement Strategies of the LOC

#### 2.3.1 Sensor‐Integrated LOC Platforms

Over the past two decades, LOC has made significant breakthroughs in vitro biomimetic modeling due to advancements in microfluidics, microengineering, cell culture technology, and materials science. These technologies have enabled LOC to accurately mimic the human liver's physiological environment, facilitating in vitro liver disease modeling and drug screening. However, most LOC systems still lack high‐precision real‐time monitoring capabilities for physicochemical parameters. Integrating sensor technology into LOC is expected to drive further development and elevate LOC technology to new heights. Physicochemical parameters in the LOC microenvironment are crucial for maintaining cell morphology, activity, and function. Real‐time observation of these parameters is essential for evaluating the chip's simulability. The main physicochemical parameters currently involved in LOC research are as follows [77, 78]:

1. LOC for real‐time oxygen metabolism monitoring

**FIGURE 8:**
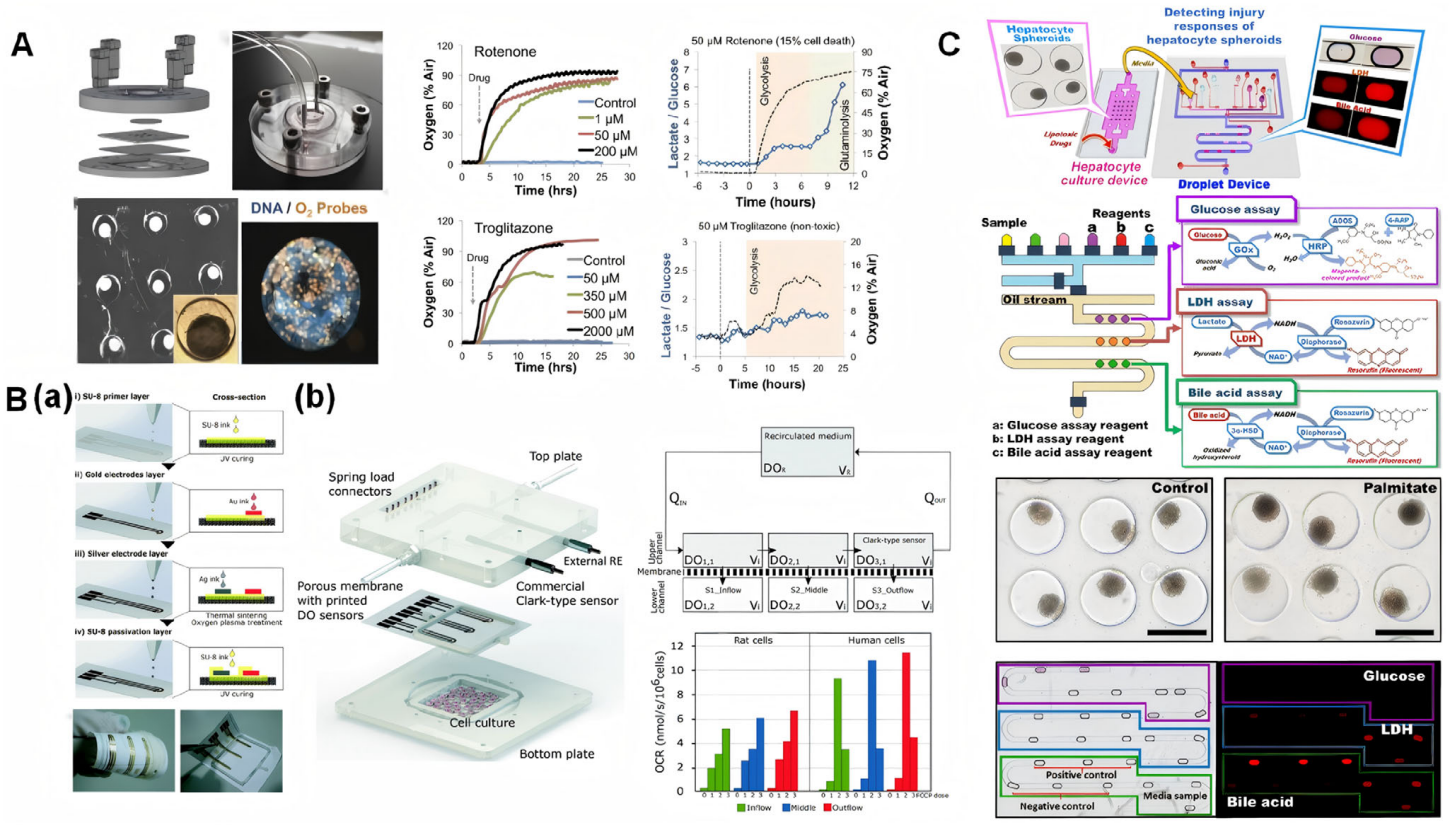
LOC platform integrating oxygen metabolism sensor and lactate‐glucose sensor. (A) Oxygen Gradient and Mitochondrial Function Monitoring in LOC‐Based Liver Models. Reproduced with permission [81]. Copyright 2016, Proceedings of the National Academy of Sciences of the United States of America. (B) (a) Fabrication steps of the DO sensor based on IJP. (b) The structure of ExoLiver platform and OCR estimation in the inflow, middle, and outflow of the bioreactor bottom channel for the stabilization period and after the three FCCP additions for rat and human hepatocyte cells. Reproduced with permission [83]. Copyright 2018, Royal Society of Chemistry. (C) Automated microfluidic platform for multi‐parameter enzymatic assays in liver injury monitoring: Illustrations of the droplet microfluidic device and its operation, and representative images of droplet‐based metabolite assays for injured hepatocytes. Reproduced with permission [84]. Copyright 2019, American Chemical Society.

Oxygen is essential for maintaining cell viability and metabolism. Oxygen consumption by cells reflects their degree of activity and metabolic status, while gradient changes in oxygen concentration mimic liver‐specific structures and functions. Therefore, accurate monitoring of oxygen is particularly important. Currently, optical sensors and electrochemical sensors have been widely used in LOC studies. Among them, mitochondria are crucial for cell maturation and metabolism. When damaged or dysfunctional, cells can switch to anaerobic pathways like glycolysis and glutaminolysis [79]. However, this damage is often unmonitored, leading to stresses such as endoplasmic reticulum (ER) stress and lipid accumulation [80]. To simulate the liver's oxygen gradient and monitor mitochondrial function in real time, Bavli et al. created a liver microarray that mimics the physiological microenvironment for long‐term HepG2/C3A cell culture (Figure 8A) [81]. They used fluorescence‐based oxygen probes to track oxygen uptake, thereby mapping mitochondrial respiration in real time, and quantified cellular adaptation to mitochondrial damage and ATP production shifts during dysfunction induced by rotenone and troglitazone (Rezulin). This system offers new insights for LOC applications in monitoring cellular metabolism and assessing drug‐induced mitochondrial changes. Inkjet printing (IJP) is a promising low‐cost printing technique to replace conventional microelectronic preparation techniques, and IJP has recently been used to integrate electrodes into microfluidic chips [82]. Moya et al. developed a modular bioreaction system called ExoLiver that mimics a liver sinusoid‐like structure. The ExoLiver consists of porous membranes separating the upper microfluidic channel and the lower static cell culture zone [83]. They used IJP technology to print electrochemical oxygen sensors and integrated them into the porous membrane, which enabled real‐time monitoring of oxygen concentration; provided real‐time information on cell status; verified the oxygen concentration gradient present in the liver structure; and investigated cellular oxygen consumption under different conditions (Figure 8B). The integration of IJP technology and OoC reduces the cost and technical difficulties of sensor integration, and also provides a reference for other printed sensors integrated with IJP chip platforms.

2. LOC integrating glucose and lactate sensors

Glucose, a key energy source, is metabolized to pyruvate under aerobic conditions and lactate under anaerobic ones. High lactate levels signal cellular stress or dysfunction. Hence, real‐time monitoring of glucose and lactate concentrations is vital for reliable and realistic organ‐on‐chip systems, particularly for assessing microenvironments, cell viability under toxins, and mitochondrial function [85]. Electrochemical sensors using glucose or lactate oxidase are common in enzyme‐based sensing. Bavli et al. developed a liver microarray model with integrated glucose and lactate sensors to detect shifts from oxidative phosphorylation to glycolysis/glutaminolysis, analyzing them alongside oxygen sensors to study mitochondrial dysfunction and its drug‐induced dynamics (Figure 8A) [81]. While electrochemical sensors are easy to integrate into microfluidic devices, they require frequent calibration and have limited lifetimes compared to optical sensors. Cedillo‐Alcantar et al. created an automated microfluidic platform using water‐in‐oil droplets for enzymatic assays, producing signals proportional to analyte concentration [84]. They validated this method by detecting glucose with a 4‐AAP/ADOS color reaction catalyzed by glucose oxidase (GOx)/horseradish peroxidase (HRP). This system was connected to a liver injury microarray model to monitor cytotoxicity (lactate dehydrogenase), energy metabolism (glucose), and liver function (total bile acids) in real‐time during palmitate‐induced liver injury. The monitoring system allows flexible fluid handling and multi‐parameter biochemical analysis with stable measurements even in trace samples (Figure 8C).

3. LOC integrating cytokine microfluidic immunobiosensors

**FIGURE 9:**
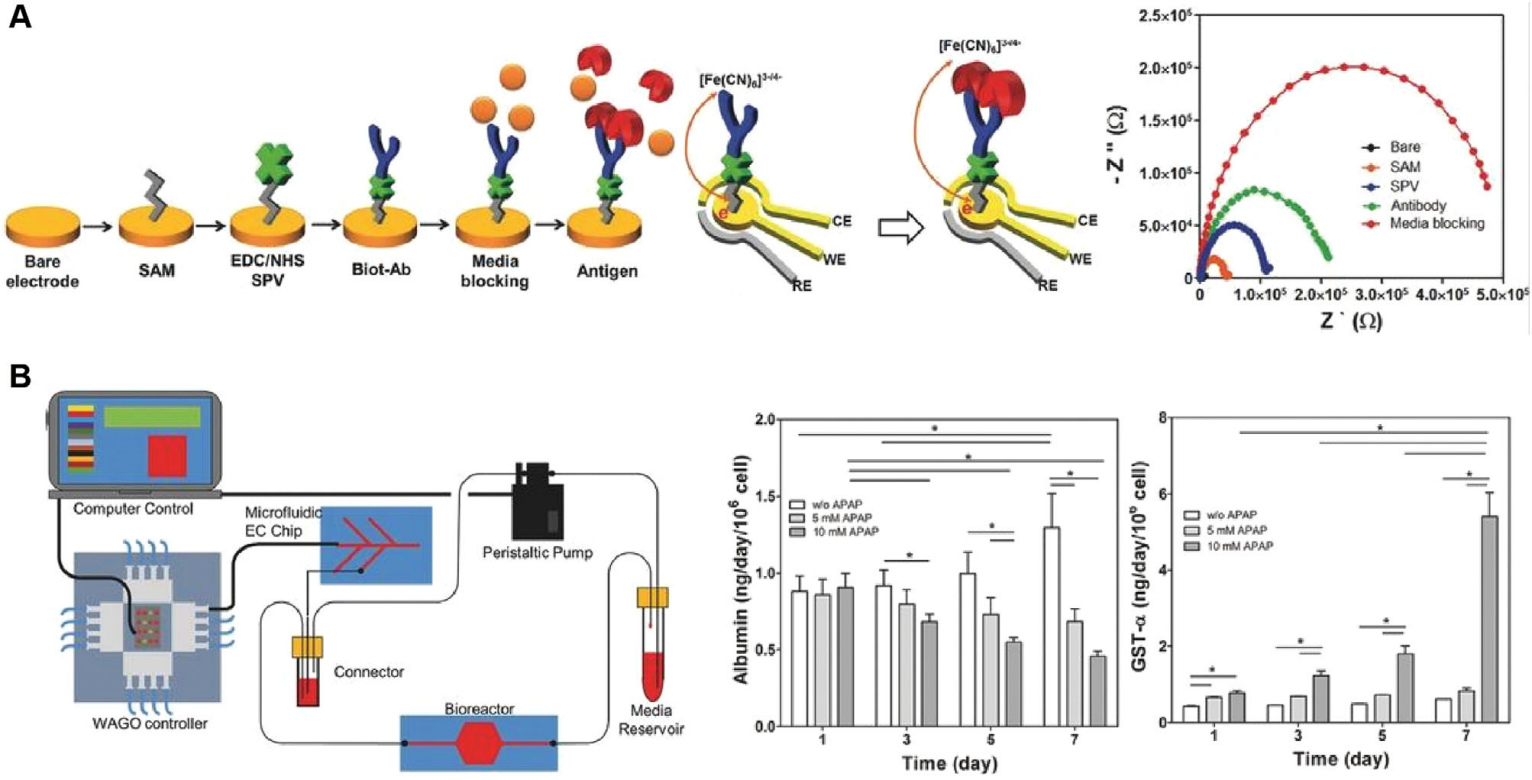
LOC integrating cytokine microfluidic immunobiosensors. (A) Detection principle of the label‐free EC biosensing system by using microelectrode and schematic illustration for immobilization of antibody using SPV on the surface of the microelectrodes and schematic of charge transfer after antigen binding upon antibody‐antigen binding for the [K_3_Fe(CN)_6_]^3−/4−^ redox process. (B) Schematic diagram of the microfluidic EC biosensing system integrated with an organ‐on‐a‐chip for continual monitoring of a target biomarker by automated manner. Continual EC measurements of albumin and GST‐α production rate in primary hepatocyte bioreactor with control under APAP exposure conditions. Reproduced with permission [90]. Copyright 2017, Wiley‐VCH Verlag.

Cytokines are pivotal in immune responses, cancer progression, and intercellular signaling; thus, real‐time monitoring of their secretion is crucial for evaluating cellular activity and function [86]. Conventional quantitative methods for cytokines include enzyme‐linked immunosorbent assays [87], aptamer‐based assays [88], and microsphere‐based immunoassays [89], but these methods are time‐intensive. Shin et al. developed a label‐free, regenerative electrochemical microfluidic immunobiosensor platform employing electrochemical impedance spectroscopy (EIS) for real‐time, label‐free monitoring of key liver biomarkers such as albumin and glutathione‐S‐transferase‐α (GST‐α) [90]. They induced hepatic metabolism with varying doses of APAP and continuously monitored cytokines within a lab‐on‐a‐chip to track biomarker alterations and cell viability in real time (Figure 9). The platform's reliability was confirmed through comparison with enzyme‐linked immunosorbent assay results. This flexible and robust microfluidic electrochemical biosensor enables automated, continuous detection of soluble biomarkers and holds promise for widespread use in drug toxicity studies and in vitro efficacy assessments.

#### 2.3.2 3D Printing‐Integrated LOC Platforms

Organ‐on‐chips are usually constructed using PDMS microfluidic devices, but the steps of traditional soft lithography are cumbersome [91]. For complex chip systems, the loading of cells and biomaterials is also complicated. 3D printing enables efficient and automated fabrication of microscale structures, enhancing the flexibility and scalability of microfluidic and organ‐on‐a‐chip production. Moreover, bioprinting technology enables precise spatial positioning of cells and biomaterials, allowing the direct construction of complex, three‐dimensional tissue‐like microenvironments within the chip. By controlling the deposition of live cells, bioinks, and matrix materials, bioprinting enhances the physiological relevance and functional performance of organ chips [92, 93].

**FIGURE 10:**
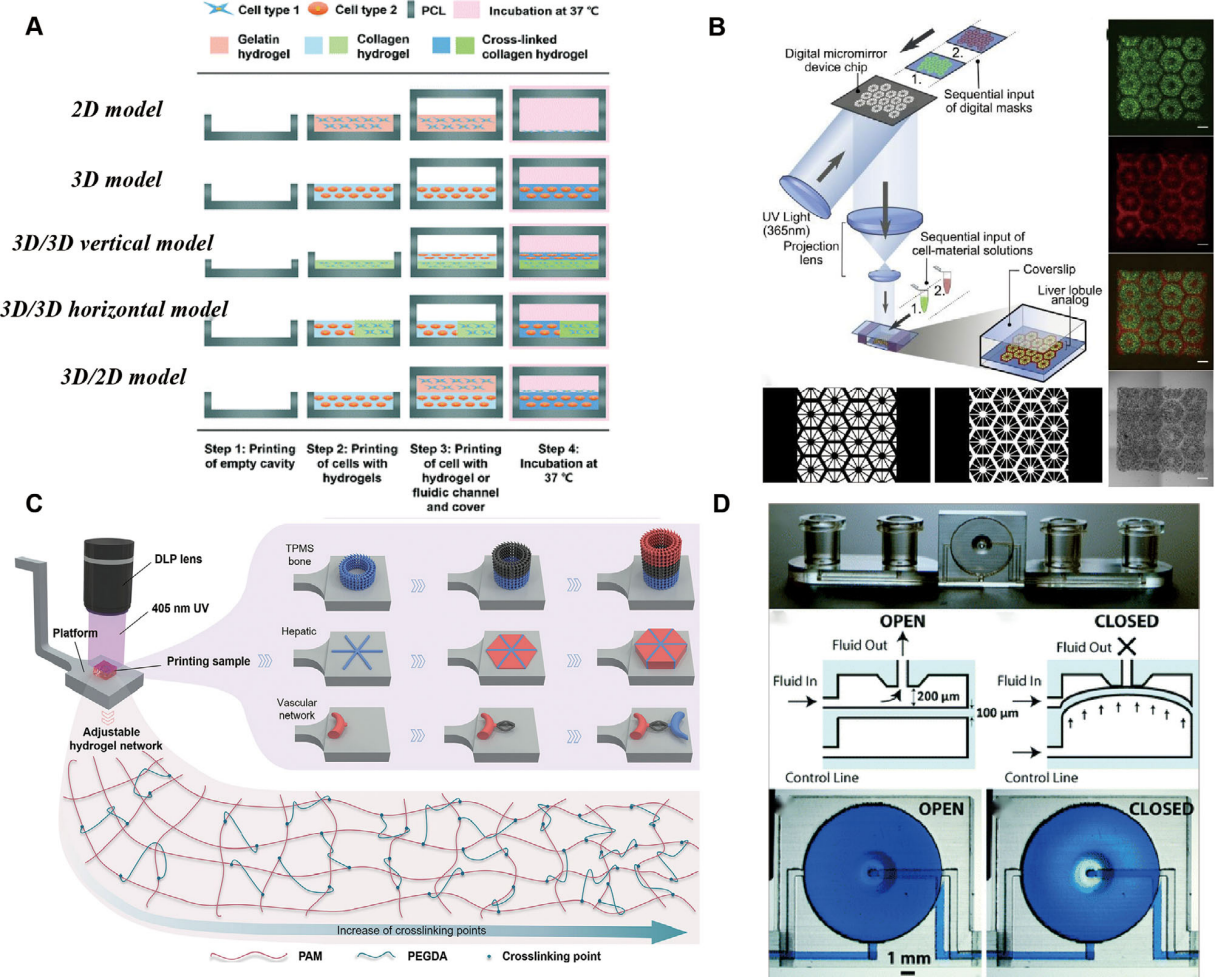
3D printing‐integrated LOC platforms. (A) Schematic of the 3D bio‐printing and the printing steps of various organ‐on‐a‐chip model images. Reproduced with permission [96]. Copyright 2016, American Chemical Society. (B) A schematic diagram illustrates a two‐step 3D bioprinting approach for fabricating hydrogel‐based hepatic constructs. Reproduced with permission [97]. Copyright 2016, Proceedings of the National Academy of Sciences of the United States of America. (C) Process illustration for multi‐material DLP 3D bioprinting of heterogeneous structures and mechanical characteristics. Reproduced with permission [98]. Copyright 2024, Wiley. (D) The design of a 3D‐printed single‐valve device. Reproduced with permission [102]. Copyright 2015, American Chemical Society.

Current integration of cells and biomaterials onto chips through 3D bioprinting typically employs two strategies. One approach involves seeding cells onto 3D bioprinted hydrogel scaffolds. For example, Zhang et al. constructed functional chips with complex perfusion networks using SLA and PEGDA, followed by GelMA grafting to facilitate HUVEC adhesion for the formation of biomimetic vasculature. This method avoids shear‐induced cell damage during printing and expands the options for bioink selection [94]. The other approach directly prints cell‐laden biomaterials to fabricate complex structures. Bhise et al. utilized GelMA to print HepG2/C3A spheroids for long‐term culture (Figure 3A), while Skardal et al. directly printed hepatocytes within UV‐crosslinked hydrogels inside microfluidic chips, forming stable liver‐like tissues that closely mimic native ECM conditions [60, 95]. Despite these advances, the direct construction of fully functional LOC systems using cell‐laden 3D bioprinting remains in its early stages. To address this limitation, Lee et al. developed a one‐step 3D printing method enabling the direct fabrication of complete chip systems, demonstrating the precise integration and localization of heterotypic cells and biomaterials within printed architectures to better replicate organ‐level physiological environments (Figure 10A) [96].

Digital light processing (DLP) technology has recently demonstrated promising applications in LOC development. Ma et al. developed a DLP‐based 3D bioprinting system for creating 3D hydrogel triple‐culture models by embedding hiPSC‐HPCs, HUVECs, and adipose‐derived stem cells into microscale hexagonal hydrogel constructs (Figure 10B) [97]. This approach promoted cellular reorganization within the constructs, enhancing the phenotype and function of hiPSC‐HPCs during in vitro culture, and confirmed the feasibility of DLP‐based bioprinting for 3D tissue models. Yang et al. further applied multi‐material DLP 3D bioprinting to fabricate PEGDA‐AAm hydrogel scaffolds with perfusable networks and tunable mechanical properties (Figure 10C). By adjusting modulus and microarchitecture, they achieved precise spatial encapsulation of multiple cell types with high biocompatibility and structural stability. For liver modelling, they developed a simplified liver lobule model with different hydrogels simulating liver plates and sinusoids, successfully localizing distinct cell types [98]. This DLP‐based approach offers a promising platform for reconstructing heterogeneous liver microenvironments and advancing drug screening and disease modelling. Applying 3D bioprinting to tissue engineering faces challenges in achieving high cell density (HCD), viability, and fine resolution simultaneously. In DLP bioprinting, light scattering reduces resolution as cell density increases. To address this, You et al. introduced iodixanol (IDX) into DLP‐based 3D bioprinting to adjust the bioink's refractive index, mitigating scattering‐induced resolution loss while enabling HCD. This strategy supports most cell types and bioinks, allowing for high cell viability and improved manufacturing resolution. The method provides valuable insights for developing bioprinting technologies that simultaneously achieve HCD, fine structural fidelity, and high cell viability, advancing the application of DLP‐based bioprinting in complex tissue engineering [99].

While many 3D bioprinting studies focus on bioreactors, scaffolds, and ECM substrates within chip systems, 3D printing is also crucial for chip fabrication. The most common method involves pouring PDMS on 3D‐printed molds for rapid microfluidic chip preparation [100]. Comina et al. developed a low‐cost micro‐stereolithography 3D printing approach to create high‐resolution (10‐50 µm) templates, thereby replacing traditional photolithography and cleanroom processes. These templates enable accurate PDMS transfer and multilayer structure fabrication, facilitating the integration of silicone tubing, varied channel heights, and 3D fluidic networks within a single print, making it ideal for rapid prototyping of complex microfluidics [101]. While PDMS valves and pumps allow fluidic automation, their fabrication typically requires advanced infrastructure and expertise. Au et al. addressed this by using stereolithography to produce the first biocompatible, transparent resin microvalves, enabling seamless integration and automated operation within microfluidic devices (Figure 10D) [102].

Advances in 3D printing and printable biomaterials have enabled efficient, automated, and modular fabrication of LOC systems at reduced cost. The integration of artificial intelligence (AI) further enhances bioprinting by autonomously learning complex structure–function relationships beyond rule‐based methods. AI‐driven approaches can optimize bioink formulations, printing parameters, and chip designs while facilitating correlation analysis between printed structures, biological activities, and host responses [103, 104]. The convergence of 3D printing, microfluidics, and AI is expected to significantly accelerate the development and biomedical applications of next‐generation LOC platforms.

#### 2.3.3 Deep Learning‐Driven LOC Platforms

**FIGURE 11:**
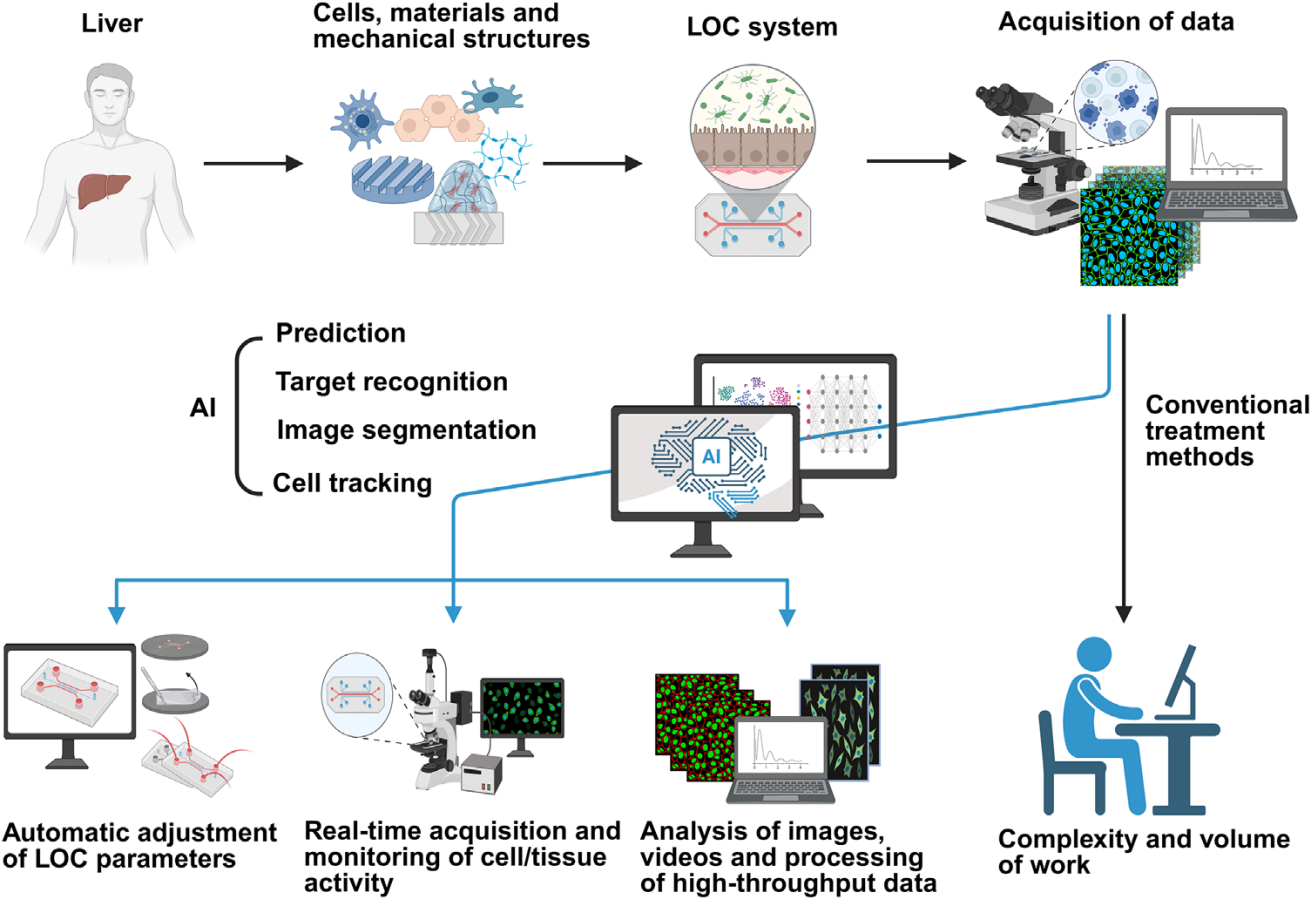
Integration of deep learning with LOC. Deep learning has been applied to device design, real‐time monitoring, and image processing with LOC (created in BioRender).

Designing microfluidic devices necessitates the meticulous determination of various parameters such as micropillars, microchannels, micropores, microchambers, fluid flow rates, shear stresses, and pressures. However, the challenge of setting and integrating these microscale parameters is exceedingly complex. OoC models offer rapid responses and high throughput, yet they generate vast amounts of data that researchers struggle to process swiftly. Consequently, there is an urgent demand for an automated, high‐throughput data analysis system capable of generating intelligent structural designs for microfluidic devices, calculating essential fluid flow rates and other conditional parameters, and aiding in data processing computations. Deep learning stands as a paramount domain within AI, distinguished for its ability to autonomously discern the inherent characteristics and patterns embedded in vast datasets, commonly referred to as “big data.” By meticulously selecting the most appropriate algorithms, this technology empowers machines to assimilate the underlying principles governing information. The integration of deep learning with OoC platforms is a new area, which holds great potential for OoC in drug screening development, disease modeling and analysis, and OoC construction. Although the integration of these two areas has not been extensively studied, applications of deep learning in OoC have been proposed for prediction, target recognition, image segmentation, and cell tracking (Figure 11) [105].

In the development of organ‐on‐chip and bioartificial organs, how to standardize the evaluation of their functional performance has been an important bottleneck restricting research progress. There are many in vitro modeling methods for liver function in the literature, covering different systems such as 2D and 3D scaffolds, spheroids, and microfluidic liver microarrays, but there is a lack of standardized indexes and cross‐platform comparability in the evaluation of functional outputs (e.g., albumin and urea secretion). In this regard, Qi's team proposed a machine learning evaluation framework, called the “3P model”, to systematically quantify the impact of construct parameters on liver functional output and predict the optimal functional maintenance time window [106]. The model integrates large‐scale experimental data from the literature, extracts and standardizes key variables such as cell source, material type, shear stress, and channel structure, and builds a functional prediction model based on XGBoost regression. Among the three modules, “Pre‐design” is used to identify key constructs, “Present” complements the secretion data curves at unreported time points, and “Prediction” further defines and calculates the functional duration (OFMD) and termination point (LFMD), providing a data‐driven decision basis for the optimal design and evaluation of organoids. The 3P model not only enhances the systematic nature and reproducibility of functional evaluation, but also provides a theoretical basis for defining non‐substantive cell participation parameters (e.g., co‐culture ratio, shear tolerance, channel structure) in future LOC designs. More importantly, the framework has good versatility and can be extended to other types of artificial organ models, promoting the evolution of organ chips from structural construction to functional standardization, and laying the foundation for cross‐platform comparison and industrial application.

Microfluidic flow sculpting is a technique used to precisely control microscale fluid flow to achieve specific fluid behaviors in microfluidic devices, which are critical for cell behavior, biochemical research, and microfabrication processes. However, the redundant variables in multidimensional space make it necessary to perform complex and large numbers of calculations to validate the results of the design. To solve the inverse setting of the design parameters problem of microfluidic devices given a desired fluid, Stoecklein et al. combined intelligent sampling and deep learning to significantly improve the efficiency and accuracy of flow‐sculpting techniques [107]. They used Convolutional Neural Networks (CNN) as the core model and designed a CNN structure containing multiple convolutional, pooling, and fully connected layers for predicting the corresponding microcolumn structure from the fluid flow image input. Then two intelligent sampling strategies, High Dimensional Model Representation (HDMR) and Principal Component Analysis (PCA), were used to collect data and evaluate the difference in model performance under different sampling strategies. Finally, the model parameters are trained and optimized, and the Pixel Match Rate (PMR) is used as an evaluation metric to measure the similarity between the predicted and target shapes. In addition, they conducted a sample generalization test to verify the reliability of the model on unknown data. This study concludes that the effectiveness of smart sampling strategies in improving the performance of deep learning models can significantly reduce the time and cost of microfluidic device design and improve the standardization, intelligence, and automation of microfluidic device manufacturing.

In cancer research, cell behavior and dynamics can reflect their role in the tumor microenvironment, and studying this interaction is important for understanding tumorigenesis and drug development. Therefore, it is particularly important to develop an automated analysis technique with high efficiency and accuracy. Mencattini et al. applied computer vision techniques and deep learning methods to study cellular dynamics in order to achieve automated high‐throughput analysis of cellular movement trajectories [108]. They first transformed the cell trajectory data into a graphical form to provide visual information input for subsequent feature extraction. Then a pre‐trained CNN was applied to extract features from the input atlas and classify them. Finally, validation tests were conducted in two types of scenarios: 3D bionic gels with immune cell‐breast cancer cell co‐culture and clustered prostate cancer cells. This approach demonstrates its potential application in the OoC field by investigating the key features extracted through deep learning. This contributes to the in vitro evaluation of cancer drug treatment efficacy, drug development, and personalized medicine.

OoC platforms often utilize time‐lapse microscopy (TLM) to capture cellular dynamics and interactions, making high spatial resolution essential for accurately recording cellular behavior. However, achieving high spatial resolution can be challenging due to physical and cost constraints. Cascarano et al. introduced a deep learning‐based Recursive Deep Prior Video (RDPV) method, which extends the Depth Image Prior (DIP) framework to enhance the super‐resolution of TLM videos without requiring any training, thereby improving operational speed [109]. When using RDPV‐enhanced TLM to observe cells, they can precisely detect cell edges, significantly reducing localization errors and enabling detailed observation of intercellular interactions. Consequently, integrating the RDPV algorithm with LOC technology in the future is expected to facilitate high‐resolution monitoring of cellular movements and interactions within the system. Currently, while there are relatively few reports on deep learning‐based LOC platforms or even OoC platforms, numerous research advancements have highlighted the significant potential of integrating deep learning with organ chip technology, as summarized below [105]:

1. Deep learning‐based organelle segmentation and tracking can generate comprehensive information about cell/tissue behaviors in OoC systems.
2. Integrating deep learning with OoC technology allows for the automatic regulation and control of various functional parameters of OoC.
3. Combining deep learning with a drug screening chip platform enables the prediction of drug toxicity through high‐throughput data analysis.
4. Real‐time physiological data analysis facilitates the study of disease development at the molecular level.
5. Combined with the human body chip, the system can construct a multi‐organ tissue model that can be analyzed by high‐throughput data analysis and achieve self‐intelligent regulation.

#### 2.3.4 Vascularized Construction of LOC Platforms

The vascular system is fundamental to maintaining the structural integrity and dynamic functionality of human organs and tissues [110]. In LOC platforms, the integration of engineered vasculature is essential not only for enhancing biomimetic fidelity but also for providing critical microenvironmental cues—such as physiological shear stress, solute gradients, and paracrine signalling—that regulate cell behavior and tissue homeostasis [111, 112]. Among the core challenges in LOC development, constructing a functional and perfusable vascular network remains a key technological enabler.

Microfluidics has become a classical means of constructing vascular pathways, offering a high degree of control over the hydrodynamic microenvironment. This method usually forms a patterned structure by soft lithography or laser etching and then lines it with endothelial cells to create a vascular‐like wall, which has the advantages of high structural reproducibility and high operational precision and is a widely used vascularization strategy in the early OoC system. Watanabe et al. successfully constructed a continuous, 3D vascular network—from microvessels to a central vein—within a microfluidic chip, offering a novel strategy for replicating the structural and functional characteristics of the liver lobule [113]. Utilizing femtosecond near‐infrared laser patterning, the researchers achieved in situ fabrication of microchannels within a hepatocyte‐laden hydrogel. By precisely optimizing laser power and incorporating a photosensitizer to enhance etching efficiency, they established a multiscale vascular network consisting of three layers of radially distributed microvessels and a vertically oriented central vein, effectively reconstructing the spatial architecture of a liver lobule–like microvascular network with high resolution and fidelity. While microfluidic techniques have enabled the precise construction of vascularized structures on chips, allowing for controlled fluid dynamics and multicellular patterning, they often face limitations in fabricating complex, hierarchical, and patient‐specific vascular architectures. To overcome these challenges and further enhance the biomimicry and scalability of vascularized chip systems, 3D bioprinting has emerged as a powerful complementary approach.

Vascularization through bioprinting is another commonly used strategy, important in that it systematically summarizes the anatomical‐physiological structure of blood vessels and realizes the structural features of blood vessels through print design [114, 115]. Constructing 3D tissues with a thickness of more than several hundred micrometers always faces the challenge of limited oxygen and nutrient diffusion, especially for liver tissues with high metabolic demand, the reconstruction of the microvascular network becomes the key to achieving tissue survival and functional expression. Liu et al. proposed and validated a strategy based on a multi‐material 3D bioprinting platform, and successfully constructed a centimeter‐sized, multi‐scale branching structure of a perfused vascularized liver tissue model. The strategy was optimized with a GelMA‐Fibrin composite hydrogel system to achieve the reconstruction of a 3D vascular network with precise structure and stable function [116]. The Qi team recently advanced vascularized chip construction by employing GelMA microgel (GMM) with 3% GelMA and 0.25% fibrin (3GF) to create composite hydrogels with microscopic heterogeneity [117]. This bioink featured tunable mechanical properties and pore structures that significantly enhanced endothelial cell spreading, invasion, microtubule formation, and nutrient transport. This strategy offers a practical approach for constructing vascularized microchip systems with immediate perfusion capability, thereby advancing functional tissue printing for organ replacement and regenerative therapies. Additionally, 3D sacrificial bioprinting remains widely utilized for fabricating hollow, perfusable vascular channels within engineered constructs. As previously noted, Massa et al. developed a 3D microvascularized liver tissue model by utilizing agarose fibers as a sacrificial template, in combination with a cell‐laden GelMA hydrogel scaffold, thereby enabling the formation of a functional microvascular network capable of sustaining perfusion [61]. They provided novel perspectives for developing 3D vascularized chips.

The construction of vascularized structures in LOC systems will be particularly crucial for advancing the emulation of hepatic sinusoidal microarchitecture, supporting sustained hepatocyte function, and enabling physiologically relevant multicellular interactions within engineered hepatic microtissues.

#### 2.3.5 Liver Buds and Organoids Integration in LOC Platforms

Liver buds are 3D hepatic precursor structures derived from the foregut endoderm. Their formation relies on complex interactions among endodermal cells, mesenchymal cells, and endothelial progenitors, representing a critical stage in embryonic liver development [118]. Liver buds possess intrinsic self‐organizing capabilities, enabling them to initiate the expression of hepatic functional markers and establish early‐stage vascular networks under defined conditions. Since Takebe et al. first reported in 2013 the generation of vascularized liver buds via co‐culture of human induced pluripotent stem cells (iPSCs) with supportive stromal and endothelial cells, liver bud engineering has gained increasing attention as a promising approach in regenerative medicine and disease modeling [18]. Liver organoids are simplified liver models generated in vitro through the induction, differentiation, and self‐organization of stem cells—either pluripotent stem cells or adult stem/progenitor cells—under three‐dimensional culture conditions [119, 120]. These organoids recapitulate partial structural and functional features of the liver and have been widely applied in drug‐induced hepatotoxicity testing and the modeling of complex liver diseases, offering great promise for translational research and organ transplantation [121, 122, 123, 124]. The integration of liver buds and organoids into LOC platforms represents a synergistic strategy combining the self‐organizing potential of developmental biology with the precise microenvironmental control of microfluidic engineering.

Unlike conventional monoculture LOC, liver buds and organoids inherently comprise multiple cell lineages and exhibit complex multicellular interactions that are crucial for liver development and function. Their integration with LOC systems is fundamentally linked to stem cell–based technologies for inducing and differentiating functional hepatic tissue [18, 125, 126]. These stem cell–driven strategies are increasingly being incorporated into microfluidic platforms, where human pluripotent stem cells (including embryonic stem cells and induced pluripotent stem cells) are sequentially guided to differentiate into hepatocytes or liver organoids [127]. When combined with biomimetic shear forces and extracellular matrix components, such systems promote cellular maturation and functional expression that more accurately recapitulate the in vivo hepatic microenvironment [126]. The study by Wang et al. proposed a novel liver organoid‐on‐a‐chip system that enables the in situ differentiation of hiPSCs into functional liver organoids within a 3D perfusable microfluidic platform. This system combines micropillar array engineering with stepwise hepatic induction, facilitating embryoid body (EB) formation, endodermal commitment, hepatic maturation, and long‐term 3D culture under dynamic flow conditions [128]. This study offers important perspectives and serves as a foundation for the continued convergence of liver organoids research and LOC platforms.

While LOC platforms are engineered based on established knowledge of organ architecture to enable precise control over cellular organization and the microenvironment, liver buds and organoids arise from intrinsic developmental programs. These stem cell‐derived constructs self‐organize into structures that recapitulate key features and functions of the native liver, offering a biologically driven complement to engineered systems [126]. However, their development is often spontaneous and difficult to regulate, limiting reproducibility and structural consistency. In contrast, LOC systems allow precise modulation of nutrient delivery, mechanical stimuli, and spatial cell arrangement. Integrating the biological complexity of liver buds and organoids with the engineering precision of LOC platforms can significantly enhance the physiological relevance and functional performance of in vitro liver models, thereby advancing applications in disease modeling, drug screening, and personalized medicine [129].

### 2.4 Application of LOC

The advent of LOC technology offers a cost‐effective alternative to animal and clinical trials for drug screening and toxicity assessments, heralding a new era in liver physiology, pathology research, and drug toxicity detection. Currently, LOC has been employed in applications such as drug hepatotoxicity testing, high‐throughput drug screening, modeling of liver tumors and other diseases, and regenerative medicine.

#### 2.4.1 LOC for Drug Screening and Toxicity Evaluation

**FIGURE 12:**
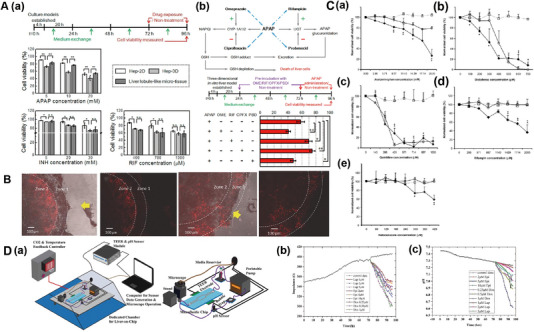
LOC for drug screening and toxicity evaluation. (A) The liver lobule chip exhibits high hepatic drug metabolism efficiency with three model drugs. (a) Drug‐induced liver toxicity of simulated tissues under APAP/INH/RIF induction. (b) Detection of drug−drug interactions via the biomimetic microtissue. Reproduced with permission [68]. Copyright 2016, American Chemical Society. (B) The dose‐dependent detection of local liver toxicity results in the APAP‐induced liver toxicity model. Reproduced with permission [40]. Copyright 2017, Wiley‐Blackwell. (C) Detection of five model hepatotoxic substances in the 3D HepaTox chip: (a) Acetaminophen, (b) Diclofenac, (c) Quinine, (d) Rifampin, and (e) Ketoconazole. Reproduced with permission [57]. Copyright 2009, Royal Society of Chemistry. (D) Advanced LOC device for real‐time hepatotoxicity monitoring. (a) Schematic diagram of a real‐time physiological sensor‐based LOC system for drug toxicity monitoring. (b) Impedance values representing the comparative cytotoxicity of epirubicin, doxorubicin and lapatinib. (c) The graph shows the comparative pH of three different cytotoxic drugs, namely epirubicin, doxorubicin, and lapatinib. Reproduced with permission [133]. Copyright 2020, IOP Publishing Ltd.

Most drugs are metabolized by enzymes highly expressed in the liver, making it susceptible to drug‐induced injury. Current in vitro and in vivo models lack the precision necessary for effective pre‐clinical evaluation of drug efficacy and toxicity before human trials [130, 131]. LOC technology addresses this gap by providing high‐precision, real‐time assessment of drug candidates toxicopathologic effects and efficacy prior to clinical trials [132]. Ma et al.’s liver lobule chip demonstrated high hepatic drug metabolism capacity under three model drugs: acetaminophen (APAP), isoniazid (INH), and rifampicin (RIF), suggesting its utility for in vitro drug toxicity assessment [68]. They also performed hepatotoxicity analysis of drug interactions, validating the bionic liver lobule microtissue for in vitro toxicology studies (Figure 12A). Weng et al. demonstrated the application of LOC in pharmacotoxic liver injury by establishing a dose‐dependent acetaminophen (APAP)‐induced hepatotoxicity model [40]. By applying various APAP concentrations to the LOC and measuring cellular activity, they observed regional hepatotoxicity not seen in conventional Petri dishes, confirming the LOC's multi‐scale functional integration and potential for translational research in clinical drug‐induced liver injury (DILI) (Figure 12B). Toh et al. used five model drugs—acetaminophen, diclofenac, quinidine, rifampicin, and ketoconazole—for hepatotoxicity assessment [57]. They employed a concentration gradient generator to deliver different drug concentrations to the cell culture area and assessed toxicity by measuring cellular activity 24 h post‐administration. Additionally, they calculated the IC50 value, the concentration producing 50% inhibition of cell activity, to verify the feasibility and practicality of the organ chip for in vitro drug hepatotoxicity testing (Figure 12C). Farooqi et al. developed a 2D monolayer LOC device with non‐invasive sensors for real‐time monitoring of transendothelial electrical resistance (TEER) and pH, demonstrating significant correlations between these parameters and cell growth dynamics [133]. This enables real‐time data on drug‐induced liver injury, aiming to create a continuous, reliable, automated sensor system that does not disturb cell growth or culture conditions. This LOC platform can accurately predict drug‐induced liver injury and hepatotoxicity, with integrated biosensors for real‐time, high‐throughput monitoring (Figure 12D).

#### 2.4.2 Liver Disease‐Related Chip

A range of LOC platforms have been established to replicate various liver diseases, encompassing alcoholic liver disease, non‐alcoholic fatty liver disease, hepatic tumors, liver fibrosis, and infectious liver disease models. These models leverage the unique capabilities of LOC technology for precise control of the cellular microenvironment, offering powerful tools for studying disease mechanisms and potential treatments.

##### 2.4.2.1 Alcoholic Liver Disease Chip

**FIGURE 13:**
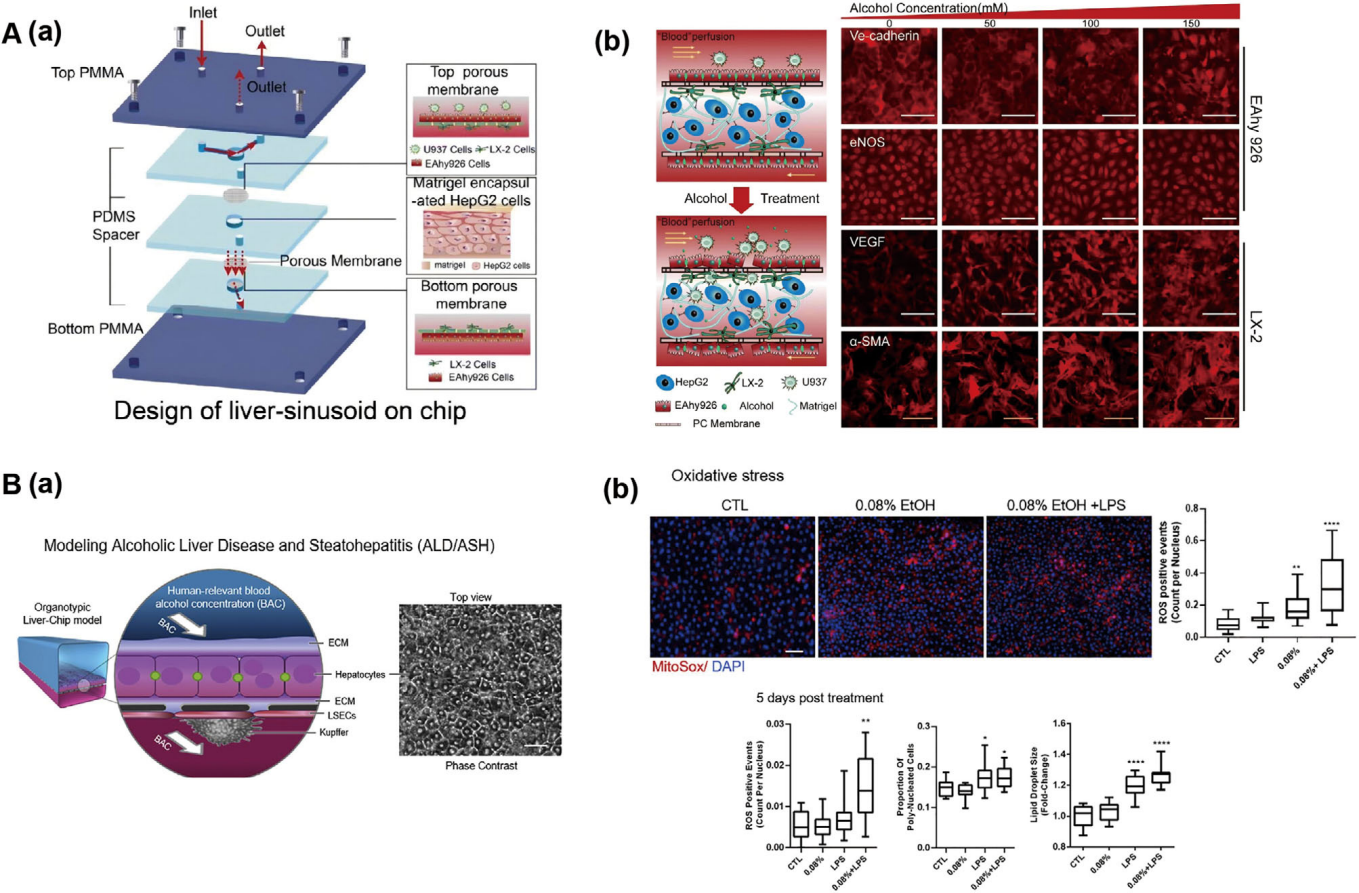
ALD‐OoC construction strategy. (A) LOC Device for Modeling NPC Roles in ALD Progression. (a) The design of the ALD‐OoC. (b) The changes in biomarkers of EAhy926 and LX‐2 cells under different alcohol concentrations in the system. Reproduced with permission [135]. Copyright 2019, Springer. (B) LOC Platform for Modeling and Alcohol Cessation Recovery analysis in Alcoholic Liver Disease. (a) Approach for modeling human alcohol‐associated liver disease/alcohol‐associated steatohepatitis (ALD/ASH) by exposing the organotypic Liver‐Chip to human‐relevant blood‐alcohol concentrations (BACs). (b) Oxidative stress detection within LOC under co‐exposure to ethanol and LPS and quantification of mean lipid droplet size and frequency of oxidative stress events and polyploidy in hepatocytes under simulated abstinence conditions. Reproduced with permission [136]. Copyright 2021, Cell Press.

Alcoholic liver disease (ALD) is a significant global health issue, causing persistent liver damage and leading to complications and mortality. Developing in vitro models that capture ALD's pathological features and reproduce its tissue microenvironment is crucial [134]. Non‐parenchymal cells (NPCs) are pivotal in ALD's onset and progression. To investigate the function of NPCs in ALD, Deng and colleagues designed a detachable LOC device [135]. This sophisticated apparatus incorporated HepG2, EAhy926, LX‐2, and U937 cells arranged to replicate the liver sinusoid architecture. Hepatocytes were sandwiched between membranes seeded with liver sinusoidal endothelial cells (LSECs) and hepatic stellate cells (HSCs), while Kupffer cells (KCs) were introduced later. This cutting‐edge model emulated alcohol‐induced damage to NPCs and aided in examining intercellular communication among diverse hepatocyte types during ALD development, utilizing biomarkers such as VE‐cadherin, eNOS, VEGF, and α‐SMA. Exposure to alcohol heightened reactive oxygen species (ROS) production, elevated VEGF levels in hepatocytes, and increased α‐SMA expression in HSCs. Additionally, it compromised tight junction integrity, diminished nitric oxide (NO) release from LSECs, activated LX‐2 cells, and resulted in an early rise in cell counts, leading to excessive extracellular matrix (ECM) secretion and fibrosis. High alcohol concentrations directly impaired LX‐2 cells, underscoring the significance of NPCs in ALD progression. The LOC device effectively simulates NPC behavior under alcohol influence, providing crucial insights for ALD pathology research and drug screening (Figure 13A).

Studies have shown that abstaining from alcohol can prevent the development of ALD. Nawroth et al. developed a LOC with bionic liver sinusoid [136]. The 3D culture setup consisted of PHHs cultured in the upper channel, and LSECs and KCs cultured in the lower channel. They modelled alcoholic fatty liver by ethanol treatment, which induces key disease features such as cellular lipid accumulation, development of oxidative stress, dysregulation of cholesterol synthesis, and alterations in the bionic bile duct network (one of the sensitive markers of liver injury). In addition, the recovery of hepatocytes after alcohol cessation was simulated. Although ethanol increased the production of ROS, oxidative stress was normalized after the recovery period (5 days of treatment with ethanol‐free medium). On the other hand, the LOC model treated with ethanol and lipopolysaccharide (LPS) to simulate more severe alcoholic liver disease did not show a decrease in ROS production after the recovery period. The model successfully simulated liver injury under alcohol‐induced injury and provided a platform for studying ALD‐related clinical features with the potential for direct translation to clinical studies (Figure 13B).

##### 2.4.2.2 Non‐Alcoholic Fatty Liver Disease Chip

**FIGURE 14:**
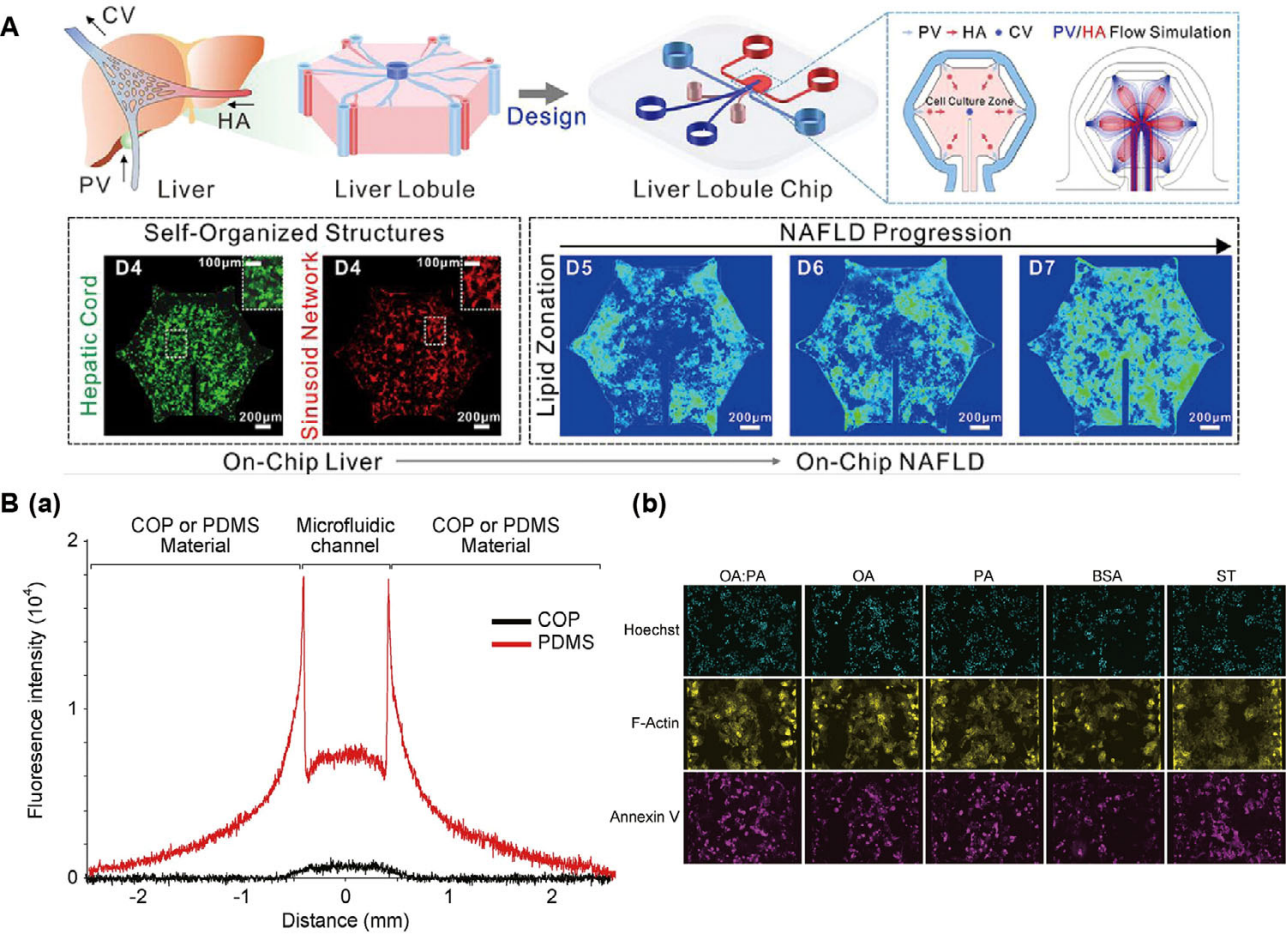
NAFLD‐OoC construction strategy. (A) Liver Lobule Chip for NAFLD Modeling and Drug Evaluation. Reproduced with permission [137]. Copyright 2021, Elsevier. (B) COP‐Based Microphysiological System for NAFLD Modeling. (a) AdipoRed fluorescent intensity profiles across the COP‐MPS and PDMS‐MPS microfluidic channels. (b) Confirmation of lipid accumulation‐induced cell death in HepaRG cells using the Annexin V fluorescent apoptosis marker. Reproduced with permission [53]. Copyright 2021, Elsevier.

The mechanisms of non‐alcoholic fatty liver disease (NAFLD) remain unclear, and effective treatments are lacking due to a lack of in vitro models that replicate the in vivo processes. Du et al. developed a liver lobule chip that simulates NAFLD by co‐culturing HepaRG cells, LX‐2 cells, and HHSECs in a scaffold‐free, three‐dimensional ECM with perfused medium exchange through hepatic artery and portal vein channels [137]. The platform's ability to mimic liver micro‐organization and function was confirmed by assessing hepatocyte albumin production and cytokine secretion. They simulated NAFLD induced by nutrient excess through the perfusion of a lipid medium, which resulted in decreased expression of both CYP1A2 and CYP3A4 under lipogenic conditions (Figure 14A). This validated the liver lobule chip for simulating liver tissue and microenvironment in NAFLD modeling. Additionally, they explored the preventive and reversible effects of obeticholic acid and flavonol on NAFLD, demonstrating the chip's potential for drug screening and therapeutic evaluation. FFAs are the key triggers of NAFLD, but PDMS‐based microphysiological systems are unsuitable for modeling NAFLD due to PDMS's absorption of hydrophobic small molecules. To enhance reliability, Wen's group proposed an in vitro NAFLD model using COP. They compared PDMS and COP microphysiological systems with AdipoRed to assess hydrophobic molecule absorption by COP [53]. HepaRG cells in the COP system were treated with FFAs to induce lipid accumulation, creating an NAFLD‐like phenotype. Apoptosis caused by lipid accumulation was confirmed with annexin V apoptosis marker, successfully establishing the in vitro NAFLD model (Figure 14B).

##### 2.4.2.3 Liver Tumor Chip

**FIGURE 15:**
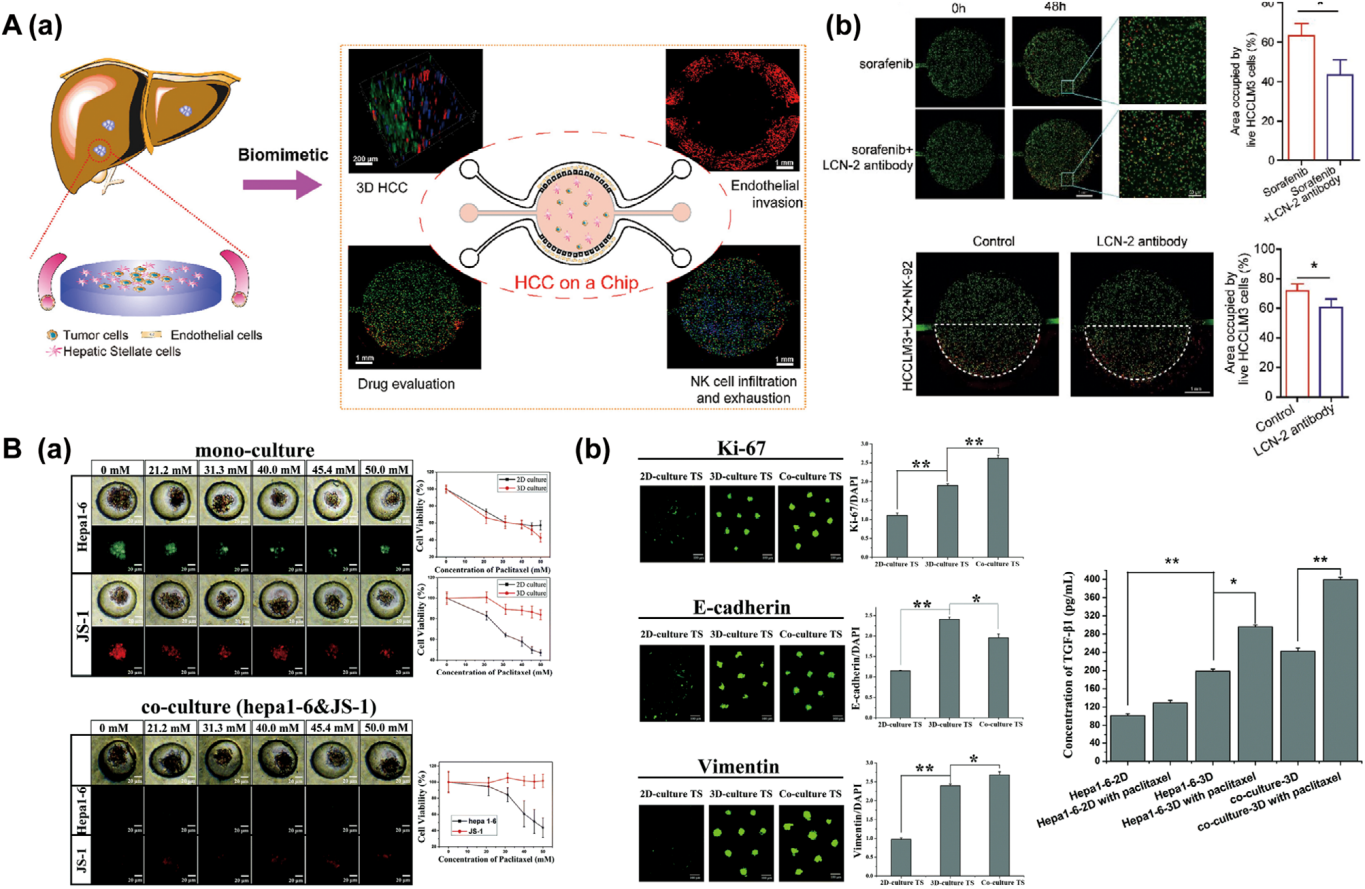
Liver tumor OoC construction strategy. (A) The liver tumor OoC paltform reveals the therapeutic potential of LCN‐2 in HCC. (a) Schematic representation of the liver tumor chip to mimic liver cancer. (b) Targeting LCN‐2 through LOC has been validated to enhance drug efficacy and reduce endothelial cell invasion. Reproduced with permission [142]. Copyright 2023, Elsevier. (B) LOC‐based 3D tumor spheroid models for hepatic tumor microenvironment and therapeutic research. Detection of drug sensitivity, epithelial‐mesenchymal transition (EMT)‐related markers, and TGF‐β1 in Hepa1‐6 tumor spheroids induced by 3D‐culturing and JS‐1 co‐culturing in the microfluidic system. Reproduced with permission [143]. Copyright 2019, Royal Society of Chemistry.

Liver cancer is one of the most prevalent primary malignancies, posing a significant threat to human health with approximately 850,000 new cases annually [138]. Consequently, the development of liver tumor models that closely mimic biological mechanisms and provide high predictive accuracy is crucial for advancing our understanding of liver cancer biology and for the development of effective antitumor therapies. To more precisely emulate the human liver tumor microenvironment (TME), Lu and colleagues developed an advanced microfluidic‐based biomimetic liver tumor microarray platform [64]. This sophisticated system combines a DLM with a GelMA hydrogel, enriched with structural proteins and growth factors, to closely replicate the natural TME (Figure 3D). By incorporating HepG2 cells into this DLM/GelMA hybrid scaffold, they effectively modeled liver tumors while maintaining higher cell viability and enhanced hepatocyte functionality compared to using GelMA alone. Furthermore, the platform's utility in drug toxicity assessment was demonstrated by testing acetaminophen and sorafenib, confirming dose‐dependent responses, validating its potential for precision oncology applications in drug screening and evaluation. HSCs are pivotal in the hepatocellular carcinoma (HCC) microenvironment, where their activation accelerates tumor progression through the secretion of ECM components and pro‐inflammatory cytokines [139]. Lipocalin‐2 (LCN‐2), a protein with high affinity for iron‐bound siderophores, has been implicated in promoting tumorigenesis by enhancing cell migration and matrix adhesion [140, 141]. In a co‐culture liver tumor model developed by Shen et al., activated HSCs were shown to contribute to tumor resistance by augmenting endothelial cell invasion and reducing NK cell‐mediated cytotoxicity towards HCC cells (Figure 15A) [142]. The study also validated the therapeutic potential of targeting LCN‐2 as an effective strategy in combating HCC. 3D tumor spheroids, formed by culturing tumor cells in vitro, recapitulate the in vivo tumor microenvironment with gradients of oxygen and nutrients, leading to necrotic cores. These spheroids maintain hepatocyte‐specific functions better than monolayer cultures. Chen et al.’s microfluidic device co‐cultured hepatic tumor spheroids with HSCs, enhancing cell interactions and establishing a TME model (Figure 15B) [143]. This setup allowed for studying cell activity, EMT factors, and drug resistance under therapeutic conditions, offering insights into liver tumor microenvironment dynamics and potential therapies.

##### 2.4.2.4 Liver Fibrosis Chip

**FIGURE 16:**
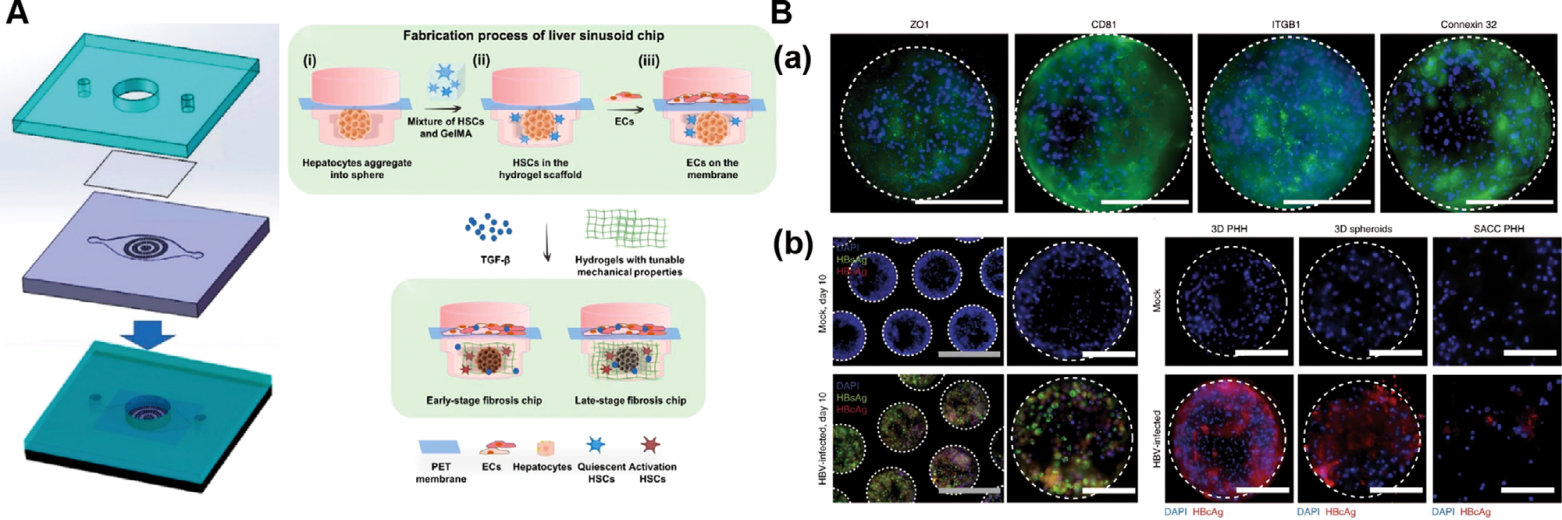
Liver Fibrosis and Hepatitis B virus infection OoC. (A) The structure and fabrication process schematic diagram of the liver fibrosis chip. Reproduced with permission [144]. Copyright 2023, American Chemical Society. (B) LOC Platform for Modeling HBV Infection and Host‐Pathogen Interactions. (a) Long‐term culture and functional tissue generation of 3D PHH cultures in a perfused bioreactor during the process of biomimetic liver tissue development. (b) Immunofluorescence detection of HBsAg and HBcAg after infection through the LOC platform, validating and emphasizing the role of NPCs in the immune response. Reproduced with permission [145]. Copyright 2018, Springer Nature.

Liver fibrosis is a progressive condition characterized by the formation of fibrous scars due to the accumulation of ECM proteins secreted by activated myofibroblasts, particularly HSCs. This process poses a significant threat to human health. However, the absence of reliable physiologically relevant liver fibrosis models has led to lengthy drug development timelines and high costs. To solve this problem, Liu et al. developed a liver fibrosis model using a PDMS‐PET‐PDMS sandwich structure with a microwell array on the bottom layer [144]. The model features endothelial cells attached to the upper membrane layer and HSCs encapsulated in GelMA, along with hepatocyte spheroids in the microwell array, mimicking the liver sinusoid and disse space. To enhance the model's reliability, they reproduced pathological features by synergizing ECM and transforming growth factor (TGF‐β). They also adjusted the porosity and mechanical strength of the matrix by varying the degree of GelMA cross‐linking to simulate different stages of liver fibrosis progression. This liver fibrosis model can replicate all stages of the disease in vitro, highlighting the importance of interactions between different hepatic cells and their microenvironment during fibrosis, allowing for the evaluation of drug efficacy at different stages, paving the way for the establishment of stage‐specific liver fibrosis models (Figure 16A).

##### 2.4.2.5 Hepatitis B Virus Infection Chip

Viral hepatitis B is a serious global health problem that can be caused by the hepatitis B virus (HBV) and may lead to cirrhosis and liver tumors. The scientific community has been facing major challenges in studying host‐pathogen interactions in viral hepatitis B due to the lack of in vitro models that can mimic the complete life cycle of HBV in hepatocytes and the growing physiologically relevant host cells that can support HBV replication. This challenge was tackled by a microfluidic platform developed by Ortega‐Prieto et al. that reproduces the entire life cycle of HBV [145]. In this system, the continuous circulation of nutrients and oxygenated medium promotes the formation of hepatocyte microtissues that remain metabolically and functionally stable for at least 40 days after inoculation, providing the conditions to study the long‐term effects of HBV on PHHs and to analyze the effects of drug therapy. It was also found that KCs, by co‐culturing with PHHs, do not specifically recognize HBV and are not involved in the early immune response, but that HBV infection induces the secretion of interleukin (IL)‐6 and tumor necrosis factor (TNF)‐α, and decreases the secretion of hepatitis B surface antigen (HBsAg) in response to stimulation with exogenous substances (Figure 16B). This model not only provides new avenues for modelling HBV‐infectious diseases and drug development but also highlights the role of NPCs in the immune response triggered by viral infection.

#### 2.4.3 Human‐on‐a‐Chip Based on LOC Platforms

**FIGURE 17:**
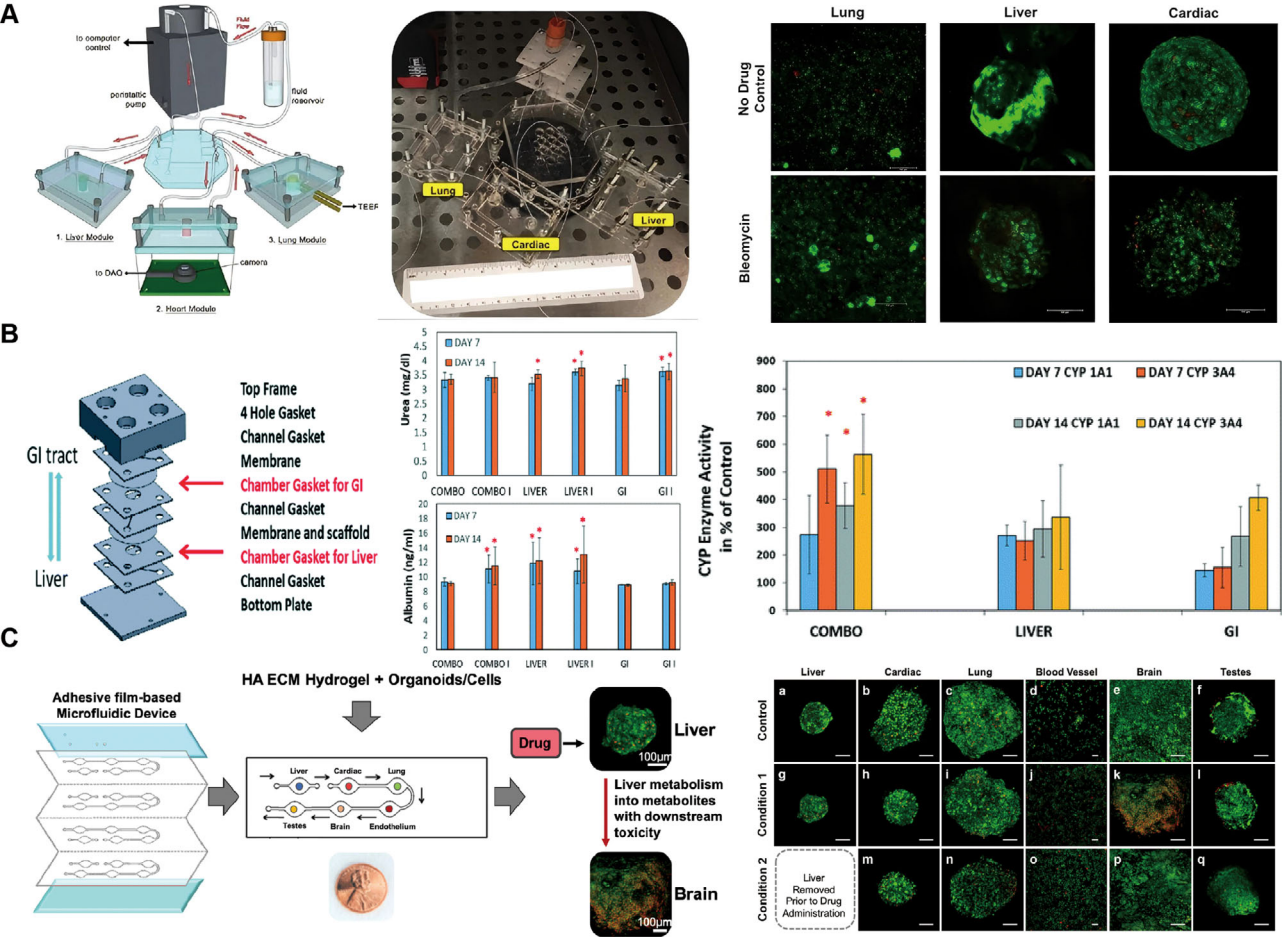
Human‐on‐a‐chip based on liver system construction. (A) Design of three‐tissue liver, heart, and lung organ‐on‐a‐chip and inter‐organ interactions and drug responses. Reproduced with permission [146]. Copyright 2017, Springer Nature. (B) The schematic of the two chambers in the GI‐Liver module and the determination of cellular metabolic rate in the presence of drugs. Reproduced with permission [147]. Copyright 2018, Royal Society of Chemistry. (C) Drug toxicity assessment of ifosfamide in a 6‐organoid system and determination of its cellular viability under conditions of injury. Reproduced with permission [148]. Copyright 2020, Elsevier.

Human organ interactions are vital for maintaining physiological functions, homeostasis, and understanding pathological conditions. Combining LOC with other organ chips creates “human‐on‐a‐chip” systems that simulate tissue‐tissue and organ‐organ interactions, reflecting human physiological environments through pharmacokinetic and pharmacodynamic analyses. These systems are crucial for modeling human drug metabolism and detecting drug toxicity. Skardal et al. developed a liver‐heart‐lung triple OoC system, integrating three organ‐specific constructs using tissue‐specific bio‐ink 3D bioprinting and microfluidic technology (Figure 17A) [146]. They applied various drugs to analyze metabolites and therapeutic/toxic effects, examining responses under multi‐organ integration. Chen et al. constructed a gastrointestinal (GI) tract‐liver system with primary human intestinal epithelial cells (hIECs) and hepatocytes, using a pumpless chip design driven by gravity to enhance metabolic activity, reduce bubble formation, increase flow rate, and lower costs (Figure 17B) [147]. This system simulates GI tract‐liver interactions, predicts drug absorption and metabolism in the GI tract, and detects hepatotoxicity, establishing physiologically based pharmacokinetic‐pharmacodynamic models. Rajan et al. extended this concept to a multi‐organ platform including liver, heart, lung, endothelium, brain, and testis, building on the liver‐heart‐lung triple OoC [148]. Their microfluidic device, made from thermoplastic polymers, features six organoid culture chambers connected via microchannels to mimic the body's basic circulation and physiology. They integrated up to twenty cell types and six organ tissues to explore drug hepatotoxicity and the effects of metabolites on downstream organs through a common circulating medium (Figure 17C). These models accurately simulate normal physiological functions of human multi‐organ systems, enabling precise in vitro drug response modelling. With high precision and accuracy, these platforms will significantly drive future drug development by assessing toxicity and efficacy.

## 3 Summary and Outlook

The evolution from 2D cell planar cultures to 3D cell cultures, and from disordered to ordered cultivation, marks significant progress in LOC research driven by advancements in microfluidic and microprocessing technologies, alongside material science. Currently, LOCs are instrumental in drug hepatotoxicity assessment, pharmaceutical screening, disease modeling, and regenerative medicine, thereby expanding the horizons of liver physiology, pathology, and drug toxicity evaluation. By integrating various single‐organ chips into a cohesive “human‐on‐a‐chip” system, these platforms enable precise and accurate evaluations of drug candidates' toxicological and therapeutic effects prior to clinical trials. The integration of sensor devices and the application of deep learning technologies further enhance these systems with capabilities for real‐time monitoring, high‐throughput data analysis, and intelligent regulation. In addition, the incorporation of 3D printing technologies offers new opportunities for fabricating highly customized and complex microfluidic architectures, enabling more precise biomimicry and rapid prototyping. Despite these advancements, the field of LOC research remains nascent, with numerous challenges yet to be addressed.

Reliable and sustainable cell sources are crucial for the construction of physiological structures and microenvironments. Stem cell‐derived hepatocytes are considered an excellent source of cells, as the pluripotency of stem cells allows them to be differentiated into desired cell types in vitro. Immature progeny can be induced to mature in specific microenvironments constructed on the system. Cells derived from patients by differentiation from stem cells also hold immense clinical value. Although PHHs are the best choice for in vitro modeling, issues such as dedifferentiation and loss of function of isolated PHHs need to be addressed. Additionally, the co‐culture model of hepatocytes and other NPCs is gaining emphasis, and reproducing cell‐cell interactions in LOC is essential for in vitro replication of structures close to the physiological microenvironment of the human body.

Over the past several years, a range of materials have been investigated and tailored for engineering LOC devices and supporting cell culture. PDMS, silicon plates, and plastics have been used as the substrate materials for most of the chips; hydrogel materials are more suitable for mimicking the natural ECM and constructing physiological microenvironments; porous PET, PC membranes, etc., are often used as the tissue‐tissue interfaces between microchannels. These materials possess attributes such as optical clarity, malleability, elasticity, permeability, stability, resistance to small molecule penetration, and biocompatibility, all while maintaining these functional properties over time. Advances in 3D printing technology have led to the development of numerous biocompatible materials suitable for bio‐3D printing and meeting LOC requirements. While these advancements in material science and fabrication techniques have propelled the progress of the LOC field, they are limited in addressing only one or a few challenges and fail to replicate the natural ECM microenvironment found in vivo, even with sophisticated construction of physiological conditions. In the control and characterization of materials for in vitro ECM construction, the integration of well‐controlled ECM into microfluidic platforms still faces great challenges, and the technology for controlling and characterizing ECM on microfluidic chips remains underdeveloped. Therefore, it is also necessary to establish a set of substrate properties, material formulations, and fabrication processes that can be used to achieve comparability and reproducibility between laboratories. Furthermore, current chip development is primarily experimental, but future commercial applications will necessitate stricter criteria regarding production costs, product reliability, and manufacturing complexity when selecting materials.

In recent years, LOC and other OoC models have been reported, but a multiplexed immune system‐on‐a‐chip has yet to be developed. The complexity of the immune system and multi‐organ linked chip conditions present challenges for the development of an immune system‐on‐a‐chip. Currently, immunomodulatory OoC primarily utilize inflammatory biomolecules, both pro‐inflammatory and anti‐inflammatory cytokines, to modulate cells and achieve the desired immune response. However, due to the high cost of these biomolecules, researchers are compelled to develop next‐generation biomaterials such as synthetic peptide structures and cell‐responsive polymers. Moreover, research on multiplexed immune system‐on‐a‐chip for drug detection remains limited, making the development of LOC with immune functionality particularly crucial. This is essential based on the normal drug delivery process in the human body, where the drug enters the bloodstream via the gastrointestinal tract and passes through the liver.

Another significant challenge lies in the process monitoring, data processing, and intelligent regulation of the system. Most LOC data are endpoint results, lacking real‐time dynamic monitoring and overlooking many key physiological processes. The specificity, rapid response, and high throughput demands of LOC place substantial requirements on data processing. While current interventions with biosensor devices and deep learning techniques are addressing these limitations, multidisciplinary approaches still necessitate extensive experimental research and validation. In conclusion, the existing LOC platforms have shown good application prospects, and the cross‐fusion of multiple disciplines and fields will bring the LOC field to a brand‐new height. We believe that more bionic and intelligent LOC systems will be put into the research of liver physiology and pathology in the future.

## Conflicts of Interest

The authors declare no conflicts of interest.
